# Clathrin mediates membrane fission and budding by constricting membrane pores

**DOI:** 10.1038/s41421-024-00677-w

**Published:** 2024-06-11

**Authors:** Lisi Wei, Xiaoli Guo, Ehud Haimov, Kazuki Obashi, Sung Hoon Lee, Wonchul Shin, Min Sun, Chung Yu Chan, Jiansong Sheng, Zhen Zhang, Ammar Mohseni, Sudhriti Ghosh Dastidar, Xin-Sheng Wu, Xin Wang, Sue Han, Gianvito Arpino, Bo Shi, Maryam Molakarimi, Jessica Matthias, Christian A. Wurm, Lin Gan, Justin W. Taraska, Michael M. Kozlov, Ling-Gang Wu

**Affiliations:** 1 National Institute of Neurological Disorders and Stroke, Bethesda, MD USA; 2 Department of Physiology and Pharmacology, Sackler Faculty of Medicine, Tel Aviv University, Ramat Aviv, Israel; 3 Biochemistry and Biophysics Center, National Heart, Lung and Blood Institute, Bethesda, MD USA; 4 Abberior Instruments America LLC, Bethesda, MD USA; 5 Department of Neuroscience & Regenerative Medicine, Medical College of Georgia at Augusta University, Augusta, GA USA; 6 Present Address: Chung-Ang University, Seoul, Republic of Korea; 7 Present Address: 900 Clopper Rd, Suite, 130 Gaithersburg, MD USA; 8 Present Address: Center of Drug Evaluation and Research, U.S. Food and Drug Administration, Silver Spring, MD USA; 9 Present Address: Emme 3 Srl - Via Luigi Meraviglia, 31 - 20020 Lainate, MI Italy

**Keywords:** Membrane fission, Endocytosis, Membrane fusion, Super-resolution microscopy

## Abstract

Membrane budding, which underlies fundamental processes like endocytosis, intracellular trafficking, and viral infection, is thought to involve membrane coat-forming proteins, including the most observed clathrin, to form Ω-shape profiles and helix-forming proteins like dynamin to constrict Ω-profiles’ pores and thus mediate fission. Challenging this fundamental concept, we report that polymerized clathrin is required for Ω-profiles’ pore closure and that clathrin around Ω-profiles’ base/pore region mediates pore constriction/closure in neuroendocrine chromaffin cells. Mathematical modeling suggests that clathrin polymerization at Ω-profiles’ base/pore region generates forces from its intrinsically curved shape to constrict/close the pore. This new fission function may exert broader impacts than clathrin’s well-known coat-forming function during clathrin (coat)-dependent endocytosis, because it underlies not only clathrin (coat)-dependent endocytosis, but also diverse endocytic modes, including ultrafast, fast, slow, bulk, and overshoot endocytosis previously considered clathrin (coat)-independent in chromaffin cells. It mediates kiss-and-run fusion (fusion pore closure) previously considered bona fide clathrin-independent, and limits the vesicular content release rate. Furthermore, analogous to results in chromaffin cells, we found that clathrin is essential for fast and slow endocytosis at hippocampal synapses where clathrin was previously considered dispensable, suggesting clathrin in mediating synaptic vesicle endocytosis and fission. These results suggest that clathrin and likely other intrinsically curved coat proteins are a new class of fission proteins underlying vesicle budding and fusion. The half-a-century concept and studies that attribute vesicle-coat contents’ function to Ω-profile formation and classify budding as coat-protein (e.g., clathrin)-dependent or -independent may need to be re-defined and re-examined by considering clathrin’s pivotal role in pore constriction/closure.

## Introduction

Membrane budding, which mediates fundamental processes such as endocytosis, intracellular trafficking, membrane-bound organelle formation, and virus infection, involves two membrane transformation steps: (1) the formation of an Ω-shaped membrane profile and (2) the cutting of the Ω-profile at the pore region (fission)^1–3^. The Ω-profile formation is accompanied by the coating of membrane proteins, among which clathrin is the most observed^4,5^, and COP I/II and caveolin are sometimes reported^2,3^. Accordingly, membrane buddings are classified as either clathrin-dependent or -independent^4–6^. Since clathrin (and other membrane coat proteins) coats Ω-profiles’ head, the primary function of clathrin (and other membrane coat proteins) is thought to be involved in Ω-profile formation, although whether and to what extent clathrin coating (and other coat proteins) provides forces to form Ω-profiles remains unsettled^4,5,7–10^. Following Ω-profile formation, proteins that form helices surrounding and constricting the Ω-profile’s pore are generally thought to mediate fission. The most commonly observed helix-forming protein for fission is dynamin and its family of proteins^11^. In brief, decades of studies establish the current view that membrane budding may require coat proteins to form Ω-profiles and helix-forming proteins to mediate fission^1,3–5,11^.

The present work examined this current view in live neuroendocrine chromaffin cells, where fission of Ω-profiles preformed via the flat-to-Ω-shape transition or formed via vesicle fusion at the plasma membrane can be readily resolved with imaging^12,13^. We found that clathrin polymerization with an intrinsically curved shape around the pore region of the Ω-profile generates elastic forces to constrict and thus close the Ω-profile’s pore. This new mechanism is of much more widespread importance than the classical role of clathrin in coating small budding vesicles because it mediates the fission of either budding or fusing vesicles of all sizes regardless of the clathrin coating on vesicles. Consequently, in addition to underlying clathrin-coated vesicle endocytosis, this mechanism underlies diverse endocytic and exocytotic modes previously considered clathrin (coat)-independent. These findings reveal a new design principle for membrane budding — the primary and universal function of clathrin and likely other protein coats with an intrinsically curved shape is to generate pore-constriction forces to mediate fission and control fusion pore dynamics. Decades of studies that classified vesicle budding and endocytosis into clathrin-dependent or -independent mode based on whether the Ω-profile’s head is coated with clathrin may need to be re-examined. Membrane fission machinery should include the intrinsically curved clathrin coat as a new class of fission proteins.

## Results

### A system to study pore closure of preformed or fusion-generated Ω-profile

**Fig. 1:**
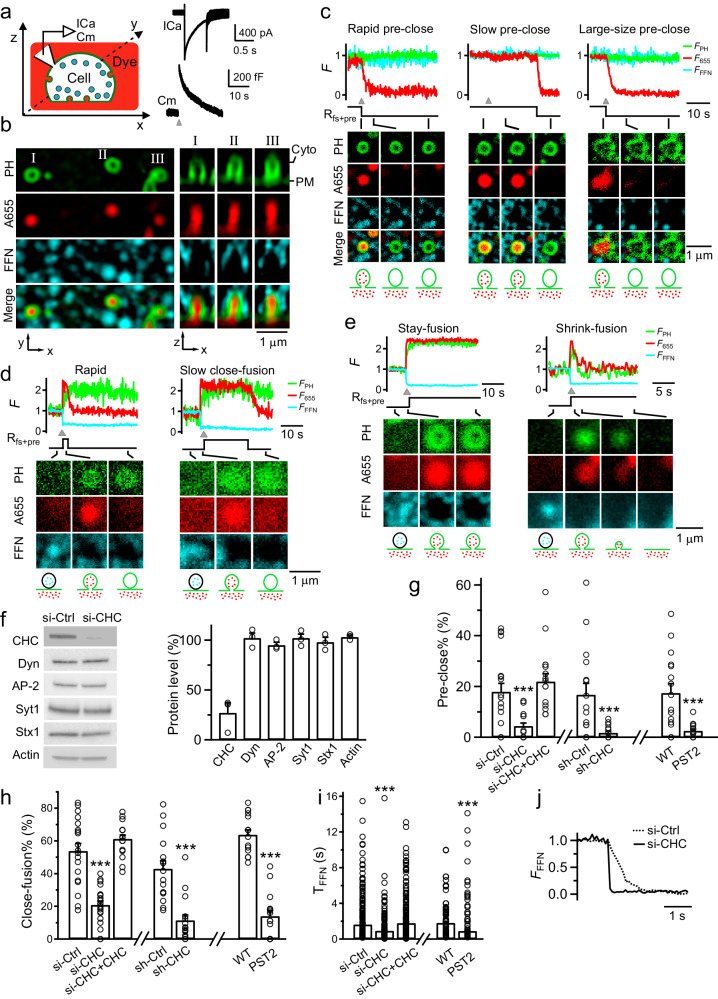
Clathrin is essential for pore closure of preformed Ω-profiles and fusion-generated Ω-profiles. **a** Left: setup drawing. The cell membrane, bath and vesicles are labelled with PH_G_ (green), A655 (red) and FFN511 (cyan), respectively. ICa and capacitance (Cm) are recorded via a whole-cell pipette. Right: sampled ICa and Cm induced by depol_1s_. **b** Confocal images of PH_G_, A655, and FFN511 (near cell-bottom) showing pre-spot I, II, and III at the XY- (left, ring-shape) and XZ-plane (right, Ω-shape). **c** PH_G_ fluorescence (F_PH_), A655 fluorescence (F_655_), FFN511 fluorescence (F_FFN_), R_fs+pre_, and sampled confocal images showing depol_1s_-induced (triangle) rapid (left), slow (middle) or large-size (right) pre-spot pore closure (pre-close). A reconstructed trace reflecting pre-close (R_fs+pre_, a down-step at F_655_ dimming onset) is also plotted. F_PH_, F_655_ and F_FFN_ were normalized to the baseline. **d**, **e** F_PH_, F_655_, F_FFN_, R_fs+pre_ and confocal images showing rapid and slow close-fusion (**d**), stay- or shrink-fusion (**e**). R_fs+pre_: a reconstructed trace reflecting fusion (up-step, at F_655_ rising onset) and fusion pore closure (down-step, at F_655_ dimming onset). **f** Left: sampled western blot results of CHC, dynamin 1/2 (Dyn), adaptor protein 2 α subunit (AP2), synaptotagmin 1 (Syt1), syntaxin 1 (Stx1), and actin in chromaffin cell cultures transfected with si-Ctrl or si-CHC. Right: the percentage (mean ± s.e.m., 3 transfections) of the above proteins in cultures transfected with si-CHC (data normalized to the corresponding mean of si-Ctrl). **g**–**i** The percentage of pre-spots undergoing pore closure (pre-close%, **g**), the percentage of close-fusion in all fusion events (close-fusion%, **h**), and FFN511 20%–80% decay time (**i**) in: (1) si-Ctrl (19 cells), si-CHC (21 cells), or si-CHC+CHC (si-CHC transfection plus CHC overexpression, 17 cells); (2) sh-Ctrl (16 cells) or sh-CHC (16 cells, FFN511 not included); and (3) Ctrl (16 cells, no pitstop 2) or pitstop 2 (PST2, 30 μM, bath, 15–30 min; 15 cells). ***P < 0.001 (*t*-test). **j** Sampled FFN511 spot fluorescence (F_FFN_) decay in si-Ctrl (dotted) or si-CHC (solid) showing faster release after clathrin knockdown.

Difficulties in separating fission from its preceding step, Ω-profile formation, have hindered the studies of fission in live cells. We recently overcame this problem by detecting pore closure of preformed Ω-profiles (pre-Ω, formed before depolarization, Supplementary Figs. S1, S2) and fusion-generated Ω-profiles (fs-Ω, Supplementary Figs. S3, S4) in live adrenal chromaffin cells^12–14^. Pre-Ω’s diameter ranges from 200–1500 nm^13^, and its body is not coated with clathrin, likely because it is too large for clathrin coating^13^. Pre-Ω could be generated from the endocytic flat-to-Ω-shape transition, including bulk endocytosis that produces vesicles larger than fusing vesicles, which may explain why a fraction of pre-Ω is larger than dense-core vesicles^13,15^; pre-Ω could be generated from dense-core vesicle fusion, some of which could maintain the Ω-shape for a long time^13^. Fs-Ω is from fusion of dense-core vesicles^12^ with a diameter of ~360 nm (range: ~200–700 nm)^16,17^; thus, Fs-Ω’s diameter ranges from 180–720 nm^12^, similar to the dense-core vesicle diameter range^16^; Fs-Ω’s body is not coated with clathrin because fusing vesicles are not coated with clathrin^12^. We used mNeonGreen attached to phospholipase C δPH domain (PH_G_, overexpressed, binds to PI(4,5)P_2_) to label the plasma membrane (PM), Atto 655 (A655, 30 μM in bath; or Atto 532) to fill Ω-profiles, and fluorescent false neurotransmitter FFN511 (or FFN206) pre-loaded into vesicles to measure release (Fig. 1a, b)^13^. At the bottom PM of the resting cell, XY-plane confocal microscopy observed FFN511-containing vesicle spots and preformed PH_G_ spots and rings overlapped with A655, but not FFN511 spots (termed pre-spot, Fig. 1b). Pre-spots were mostly pre-Ω (88 ± 3%, 13 cells; width: ~200–1500 nm) with a pore between 0–630 nm, but sometimes Λ-shaped, as observed at the XZ-plane with confocal or stimulated emission depletion (STED) microscopy (e.g., Fig. 1b; Supplementary Fig. S1; for detail, see ref. ^13^). A whole-cell 1-s depolarization (–80 to +10 mV, depol_1s_) induced calcium currents (ICa), capacitance changes (reflecting exo-endocytosis, Fig. 1a), pre-spot closure (pre-close, Fig. 1c), and fusion spots observed with confocal microscopy (Fig. 1d, e; cell-bottom, XY-plane imaging every 40–80 ms)^12–14^.

Pre-close was detected as A655 fluorescence (F_655_, strongly excited) dimming while PH_G_ fluorescence (F_PH_, weakly excited) sustained or dimmed with a delay (Fig. 1c)^13^. This method detected pore closure that was impermeable to H^+^ and OH^–^, mediated by dynamin, and observed directly with STED imaging (e.g., Supplementary Fig. S2)^12–14,18^. Pre-close reflected mostly pre-Ω closure, as preformed-Λ closure was negligible^13^. It retrieved ~200–1500 nm vesicles (~17%: 600–1500 nm, e.g., Fig. 1c)^13^.

Fusion spots were detected as a sudden appearance of PH_G_ and A655 spots while FFN511 spot fluorescence (F_FFN_) decayed, due to the diffusion of PH_G_/A655 from the PM/bath to the fs-Ω and release of FFN511 from the fs-Ω (Fig. 1d, e). STED imaging directly observed these diffusion events (e.g., Supplementary Figs. S3, S4; for detail, see refs. ^12,13,19^). Three fusion modes were observed (see Materials and methods for more detail): (1) close-fusion (kiss-and-run)–fs-Ω pore closure was detected similarly to pre-close: as F_655_ dimming while F_PH_ was sustained or decayed later (Fig. 1d)^12–14,18^; (2) stay-fusion — a sustained fs-Ω was detected as persistent PH_G_/A655 spots with sustained F_655_ and F_PH_ (Fig. 1e); (3) shrink-fusion–fs-Ω shrinking was detected as parallel decreases of spot-size with F_655_ and F_PH_ (Fig. 1e)^12,18,19^. STED imaging directly observed these modes (e.g., Supplementary Fig. S4; for detail, see refs. ^12,19^).

### Clathrin is required for pre-Ω and fs-Ω pore closure

Three sets of data suggested that clathrin is required for pre-close and close-fusion. First, clathrin heavy chain (CHC) siRNA (si-CHC) transfection, which reduced CHC without affecting dynamin or other proteins (Fig. 1f), substantially reduced depol_1s_-induced pre-close (Fig. 1g), close-fusion (Fig. 1h), and FFN511 20%–80% decay time (T_FFN_-release time, Fig. 1i, j). CHC overexpression in si-CHC-transfected cells rescued pre-close, close-fusion, and T_FFN_ to control level (Fig. 1g–i). Second, CHC shRNA (sh-CHC) transfection, another knockdown approach that reduced immuno-labeled clathrin puncta (Supplementary Fig. S5), substantially reduced pre-close and close-fusion (Fig. 1g, h) detected by imaging with A655 and Alexa 488 without PH_G_ (Supplementary Fig. S6, see also Materials and methods and refs. ^18,20^). Third, clathrin inhibitor^21^ pitstop 2 substantially reduced pre-close (Fig. 1g), close-fusion (Fig. 1h), and T_FFN_ (Fig. 1i), but not dynamin expression (Supplementary Fig. S7). Since fs-Ω pore constriction/closure competes with pore expansion to determine pore dynamics^12^, blocking fs-Ω closure may generate larger fusion pores^12^, which explains the T_FFN_ reduction shown in Fig. 1i.

### Clathrin is at Ω-profiles’ base/pore region for pore constriction and closure

The requirement of clathrin for pre-Ω and fs-Ω pore closure implies that clathrin acts at the pore region. The following three sets of data showed that clathrin is at the Ω-profile’s base/pore region to mediate pre-Ω and fs-Ω pore closure.

**Fig. 2:**
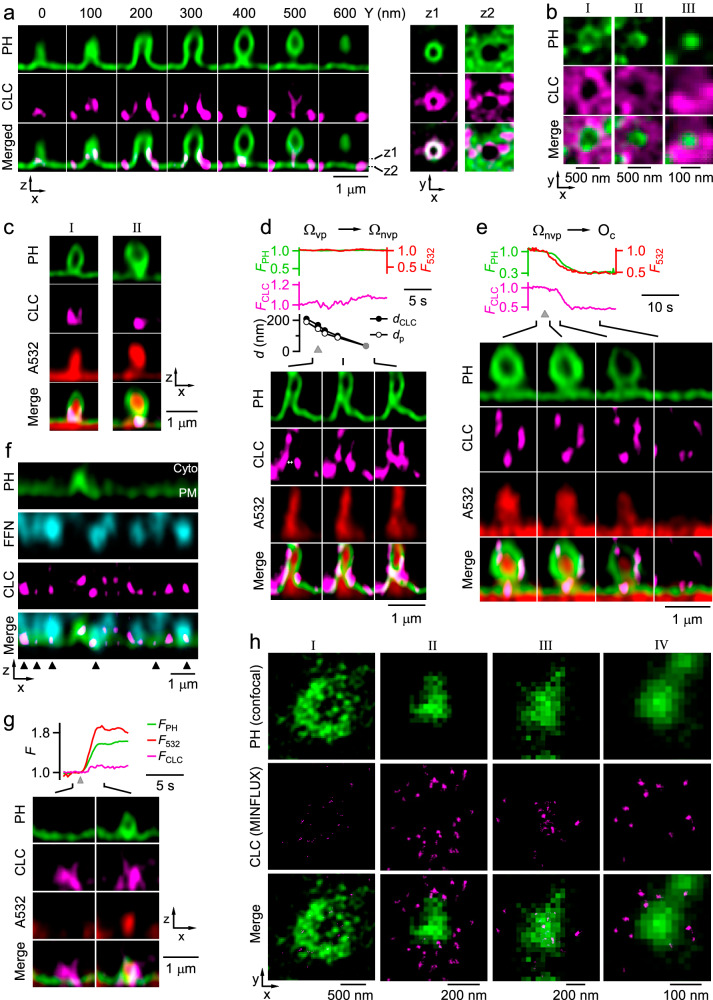
Clathrin at Ω-profile’s base/pore region constricts the pore. **a** Left: clathrin surrounds pre-Ω’s visible pore: STED XZ-plane images of PH_G_ and CLC-mTFP1 for a pre-Ω with a visible pore (pre-Ω_vp_) along *Y*-axis every 100 nm as labelled. Right: XY-plane images at two Z-planes (z1, z2) across the pore region. **b** STED XY-plane images of PH_G_ and SNAP-CLC-SiR (see Materials and methods) showing different sizes of pre-Ω’s pore surrounded by clathrin (left: pore visible; middle and right: pore too small to resolve). **c** Clathrin at pre-Ω’s non-visible pore and base region: STED XZ-plane images of PH_G_, CLC-mTFP1 and A532 for a pre-Ω with a non-visible pore (pre-Ω_nvp_, permeable to A532). **d** CLC-mTFP1 puncta flanked and moved with the constricting pore of a pre-Ω_vp_: F_PH_, F_532_, CLC-mTFP1 fluorescence (*F*_*CLC*_), Ω_p_’s pore diameter (*d*_*p*_), the distance between two CLC-mTFP1 puncta flanking the pore (*d*_*CLC*_), and sampled STED XZ-plane images of PH_G_/CLC-mTFP1/A532 taken at times indicated sticks. Gray circles: < 60 nm (STED resolution); triangle: depol_1s_. **e** CLC-mTFP1 puncta at the base/pore region of a pre-Ω_nvp_: F_PH_, F_532_, F_CLC_ and sampled STED XZ-plane images of PH_G_/CLC-mTFP1/A532. F_532_ decay (A532 was strongly excited) reflected pre-Ω pore closure. Triangle: depol_1s_. **f** STED XZ-plane images of PH_G_, FFN206, and CLC-mTFP1 (merge at the bottom) showing CLC-mTFP1 puncta at vesicle docking sites (triangles, contact between FFN511-labelled vesicles and the PH_G_-labelled PM). PM and cytosol (Cyto) locations are labelled. **g** F_PH_, F_532_, F_CLC_ (F) and sampled XZ/Y_fix_ images of PH_G_/A532/CLC-mTFP1 showing that PH_G_/A532-labelled fs-Ω co-localized with pre-existing CLC-mTFP1 puncta at fs-Ω’s pore region (8 out of 8 fs-Ω). **h** Confocal images of PH-mScarlet showing the visible (I) or non-visible pore (II–IV) of 4 Ω-profiles at the XY-plane (upper) and 2D MINFLUX images of CLC-SNAP-A647 at the same XY-plane region (middle, upper and middle images merged at the bottom).

First, volume scanning (XZ-plane scanning alone Y-axis every 100–200 nm for 5–10 μm or XY-plane scanning alone Z-axis every 100–200 nm for 2 μm across the cell-bottom membrane) showed that clathrin light chain (CLC, attached with mTFP1 mostly, but sometimes a SNAP-tag) was associated with or surrounded most PH_G_-labelled and Atto 532 (bath)-filled pre-Ω’s visible (Ω_vp_, > ~60 nm) or non-visible (Ω_nvp_, < ~60 nm, STED resolution) pore and base (124 out of 139 pre-Ω, 19 cells, Fig. 2a–c; see also Fig. 2d, e), suggesting that clathrin is available at the pre-Ω’s pore region to mediate pore closure. Furthermore, most FFN206-labelled vesicles docked at PH_G_-labelled PM colocalized with CLC-mTFP1 puncta at vesicle-PM contact zones (96 ± 2%, 5 cells; Fig. 2f), suggesting that clathrin is available at the fusion site to mediate fs-Ω’s pore closure.

Second, we applied 1 depol_1s_ per cell to induce pore closure while repeatedly performing the XZ-plane STED imaging at a fixed *Y*-axis location (near the cell center) every 50–300 ms (XZ/Y_fix_ scanning), which improved time resolution and thus allowed us to observe pore closure in real-time but reduced the chance of seeing Ω-profiles^12,13^. 3-color STED XZ/Y_fix_ scanning showed that CLC-mTFP1 puncta were at the base/pore region during pre-Ω_nvp_ pore closure (Fig. 2e, 12 out of 13 events, 13 cells), that pre-Ω_vp_ pore constriction paralleled the decrease of the distance between CLC-mTFP1 puncta flanking pre-Ω_vp_’s pore (Fig. 2d, *n* = 3 events, 3 cells), and that depol_1s_-induced, PH_G_/Atto 532-labelled fs-Ω (ref. ^12^) colocalized with pre-existing CLC-mTFP1 puncta at fs-Ω’s pore region (8 out of 8 fs-Ω, 8 cells; Fig. 2g). These results showed in real time that clathrin is at or surrounds the base/pore region to mediate pore closure during pre-Ω and fs-Ω pore closure.

Third, to examine clathrin distribution in pre-Ω’s pore region in more detail, we performed minimal photon fluxes (MINFLUX) imaging of CLC attached with a SNAP-surface® Alexa fluor 647 (SNAP-CLC-A647) at a localization precision of ~3 nm^22^. We first performed XZ-plane confocal imaging of PH-mScarlet (similar to PH_G_, except mNeonGreen replaced with mScarlet) along *Y*-axis every ~0.5–1 μm (XZ/Y_stack_ scanning) to identify the pre-Ω’s pore region, which appeared mostly as confocally non-visible pore, a PH-mScarlet spot (Fig. 2h_II-IV_), but occasionally confocally visible as a ring at the XY-plane (Fig. 2h_I_) (see also Fig. 2b for STED images). These structures could also be observed confocally at the XZ-plane (Supplementary Fig. S8, see also Fig. 2a, c for STED images). Next, we performed 2D MINFLUX imaging of SNAP-CLC-A647 at these identified pre-Ω’s base/pore region at the XY-plane (Fig. 2h). MINFLUX imaging resolved many individual SNAP-CLC-A647 molecules as clusters of localizations around or at the pre-Ω’s pore region (22 pre-Ω, 17 cells, 4 cultures; Fig. 2h). Some SNAP-CLC-A647 molecules were localized close together within a ~10–30 nm distance (Fig. 2h), consistent with clathrin polymerization in these regions^4,5^.

### Clathrin polymerization is required for pore closure

**Fig. 3:**
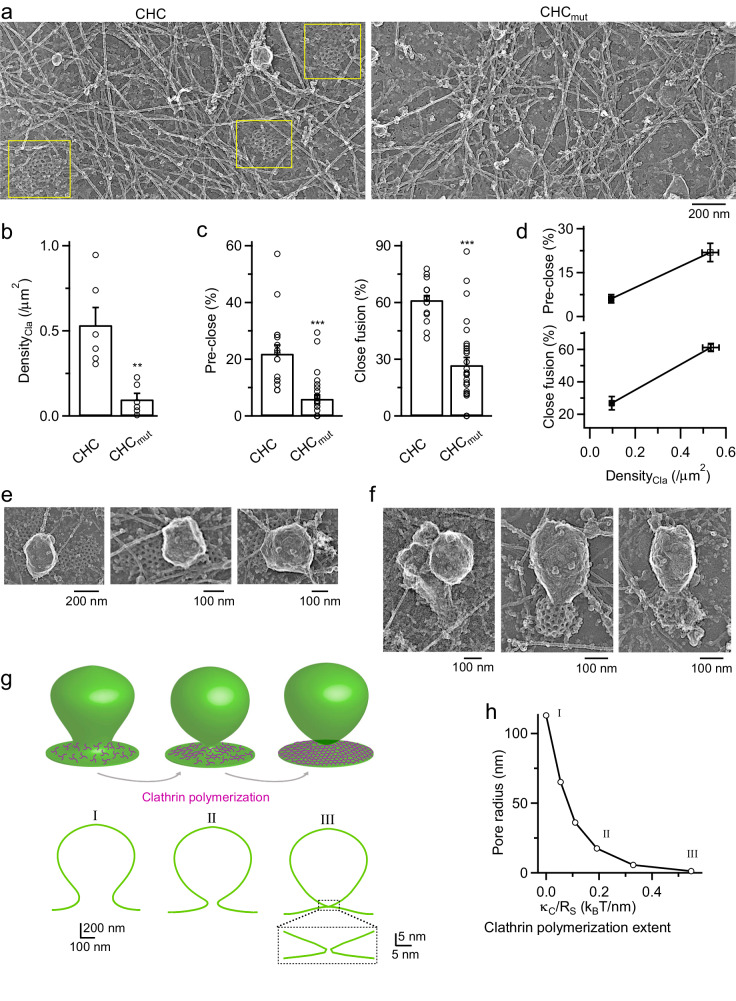
Clathrin polymerization at Ω-profile’s base/pore region produces forces to constrict and close the pore. **a**, **b** Platinum replica EM images (**a**) and clathrin patch density (Density_Cla_, **b**, mean + s.e.m.) in cells transfected with si-CHC plus wildtype CHC (si-CHC + CHC, abbreviated as CHC; 6 cells) or C1573S CHC mutant that prevents clathrin trimerization (si-CHC+CHC_mut_, abbreviated as CHC_mut_, 6 cells). Yellow squares in panel **a** indicate clathrin patches. Clathrin patch density was plotted in panel (**b**). **c** Pre-close% and close-fusion% (mean + s.e.m.) in cells transfected with si-CHC+CHC (CHC, 17 cells) or si-CHC+CHC_mut_ (CHC_mut_, 28 cells). **d** Pre-close% and close-fusion% (mean + s.e.m.) plotted versus Density_Cla_ (mean + s.e.m.) from cells transfected with si-CHC+CHC or si-CHC+CHC_mut_ (data obtained from panels **b** and **c**). **e** Platinum replica EM images from control cells: clathrin lattices are associated with oval-shape membrane structures (top-to-bottom view). **f** Platinum replica EM images showing clathrin lattices are associated with the apparently tilted oval-shaped membrane structures at the base/pore region. **g** Evolution of Ω-bud shape (including pore waist). Upper row illustrates the extent of clathrin polymerization at the base; lower row presents the computed shape profiles. Panel I: no clathrin polymerization; panel II: partial clathrin polymerization; panel III: full clathrin polymerization. The degree of clathrin polymerization is quantified by the value of torque, ${\kappa }_{C}/{R}_{S}$, indicated in panel **f** by the numbers corresponding to those at the panels. Coat area is kept constant (= $\mathrm{302,191}{\rm{n}}{{\rm{m}}}^{2}$). Ω-bud’s height: 1128 nm; $\Omega$-bud’s base boundary width: 282 nm; from typical $\Omega$-buds observed in chromaffin cells^13^. Panel III inset: boxed area showing enlarged pore waist region with a pore radius of 1.2 nm for a fully polymerized coat. **h** Pore radius plotted versus ${\kappa }_{C}/{R}_{S}$ quantifying the extent of the coat polymerization. Data points I–III correspond to Ω-bud profiles I–III in panel **g**, respectively.

Three sets of evidence suggest that clathrin polymerization is needed for pore closure. First, pitstop 2, which blocks dynamic clathrin patch formation and disassembly^21^, inhibited pre-Ω/fs-Ω closure (Fig. 1g, h). Second, platinum replica electron microscopy (EM) in si-CHC-transfected cells showed that overexpression of C1573S CHC mutant, which abolishes clathrin trimerization^23^, reduced clathrin lattice density when compared to wildtype CHC overexpression (Fig. 3a, b). Consistently, pre-close and close-fusion percentages in si-CHC-transfected cells overexpressed with C1573S CHC were much lower than those in si-CHC-transfected cells overexpressed with wildtype CHC (Fig. 3c). Evidently, lower clathrin lattice density was associated with lower pre-close and lower close-fusion (Fig. 3d). These results suggest that clathrin polymerization is required for mediating pre-Ω/fs-Ω pore closure. Third, a top-to-bottom EM view showed that clathrin lattices associated with some apparently oval/round shape membrane invaginations (9.1 ± 1.2%, *n* = 20 cells; Fig. 3e) reminiscent of STED images of clathrin surrounding pre-Ω’s base/pore region (Fig. 2a–c). Invaginations not associated with clathrin lattices could be because: (1) a top-to-down view precludes seeing Ω-profiles’ base/pore region, and (2) EM may not recognize non-polymerized clathrin. While most Ω-profiles were apparently oriented in parallel to the top-to-down direction, which showed a complete oval/round-shape bright and clear edge (Supplementary Fig. S9a; see also Fig. 3e), some Ω-profiles were apparently tilted — a U- or C-shape bright and clear edge plus the remaining non-bright and non-clear edge that appeared to be connected with the plasma membrane (Supplementary Fig. S9b; see also Fig. 3f). In 24% (16 out of 66) of these apparently tilted Ω-profiles (20 cells), we observed clathrin lattices at the non-bright and non-clear edge, the base/pore region of the apparently tilted Ω-profiles (Fig. 3f), supporting that clathrin may polymerize at the Ω-profile’s base/pore region.

### Modelling: clathrin polymerization generates pore-closure forces

We performed theoretical modeling to determine how clathrin polymerization generates pore-closure forces. Our modeling started with a Ω-bud without clathrin as recently computed^9^; clathrin was then recruited to the Ω-bud’s base and gradually polymerized into a coat. The coat was assumed to have an inherent stress-free conformation of an element of the elastic sphere with bending modulus, ${\kappa }_{C}$, and intrinsic radius of curvature, ${R}_{S}$ (Fig. 3g, h; see Materials and methods). The coat then applied a torque to the Ω-profile’s base. Torque value reflected coat polymerization extent, ranging from 0 before polymerization to ${\kappa }_{C}/{R}_{S}$ for complete polymerization, where ${R}_{S}=70{\text{nm}}$, a typical clathrin-coated vesicle’s radius^7^, and the reported ${\kappa }_{C}$ is approximately equal or significantly the membrane bending modulus ${\kappa }_{\text{m}}\approx 20{\text{ k}}_{\text{B}}\text{T}$ ${(\text{k}}_{\text{B}}\text{T}=4.1{10}^{-21}\text{Joule}$, product of Boltzmann constant and absolute temperature)^24,25^.

Computation (see Materials and methods) revealed that as ${\kappa }_{C}/{R}_{S}$ (the polymerization extent) increases, Ω-bud’s pore gradually constricts from ~110 nm (non-polymerized clathrin) to nanometers (fully polymerized coat, Fig. 3g, h). For ${\kappa }_{C}=40{ k}_{B}T$ (ref. ^24^) and ${R}_{S}=70\text{ nm}$ (ref. ^7^), pore radius reached ~1.2 nm (Fig. 3g, h), which may lead to fission^26,27^. For a larger reported ${\kappa }_{C}$ (refs. ^24,25^), the pore radius became even smaller, but beyond the model validity. Thus, the intrinsically curved shape of a rigid clathrin coat covering the Ω-bud base may constrict and close the pore. This conclusion was obtained with the constant area scenario for clathrin coat formation^28^, consistent with the observation of clathrin at the pre-Ω’s base/pore region (Fig. 2a–e). A similar conclusion was obtained for the constant curvature scenario^28^ (Supplementary Fig. S10).

### Clathrin is essential for clathrin-coated vesicle pinch-off

**Fig. 4:**
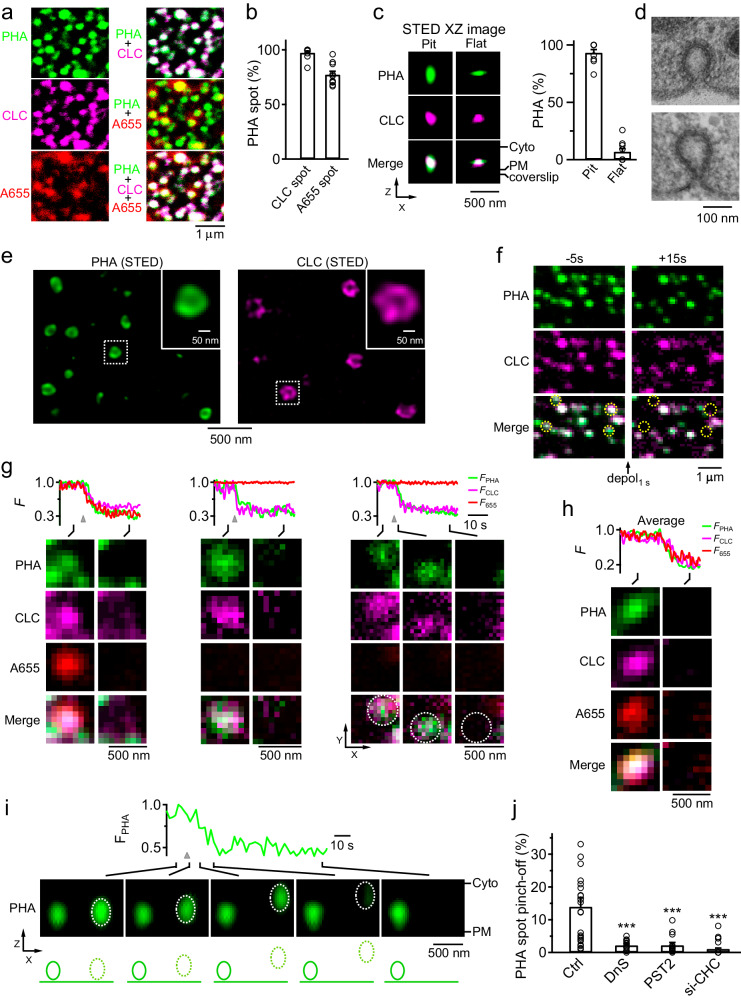
Clathrin-mediated pore closure underlies clathrin-coated vesicle pinch off. **a** Confocal XY-plane PHA_G_, CLC-mTFP1, and A655 images (left) and their merged images (right). Since images were obtained by averaging as much as 128 times, these puncta were not visible in experiments shown in Figs. 1, 2. **b** The percentage of PHA_G_ puncta colocalized with CLC-mTFP1 or A655 spots (10 cells). **c** Left: STED XZ-plane PHA_G_ and CLC-mTFP1 images (merged at bottom) showing an oval- (pit) and a flat-shape spot (STED resolution: X: ~ 60–80 nm; Z: ~ 150–200 nm). Right: percentage of PHA_G_ spots showing oval/round- or flat-shape (mean + s.e.m., 8 cells). **d** EM images showing two typical clathrin-coated pits in chromaffin cells. **e** STED XY-plane images of SNAP-tag-labelled PHA (SNAP-PHA-SiR, left) or CLC (SNAP-CLC-SiR, right) show rings resembling clathrin-coated pits. The boxed areas are enlarged in the insets. Images were obtained with a higher-spatial-resolution (~30 nm, XY-plane) STED scope equipped with a high-power 775-nm depletion laser (see Material and methods for more detail). **f** Confocal XY-plane PHA_G_ and CLC-mTFP1 images before (–5 s) and after (+15 s) depol_1s_. Dotted circles indicate PHA/CLC puncta pinch off after depol_1s_. **g** Fluorescence (F) of PHA_G_ (F_PHA_), CLC-mTFP1 (F_CLC_), and A655 (F_655_) and sampled confocal XY-plane images showing three PHA/CLC puncta pinch off (one with a A655 spot, two without A655 spots). Dotted circles indicate PHA/CLC puncta XY-plane movement after pinching off. **h** F_PHA_, F_CLC_, F_655,_ and sampled confocal XY-plane images showing an averaged PHA/CLC/A655 spot pinch-off. The images were aligned at the onset of F_PHA_ decay. **i** F_PHA_ and confocal XZ-plane images showing a PHA_G_ spot (right, dotted) moving from the PM towards cytosol after depol_1s_ (gray triangle). Another spot (left) did not move, serving as control. **j** The percentage of PHA_G_ spot pinch-off in the absence (Ctrl, 24 cells) or presence of dynasore (DnS, 80 μM; 30 min, bath; 14 cells), pitstop 2 (PST2, 30 μM, bath, 10–30 min, 16 cells), or si-CHC (12 cells).

Clathrin underlies pore closure of ~200–1500 nm pre-Ω/Fs-Ω (Fig. 1), which is too large for clathrin to coat the Ω’s head (Fig. 2, see also ref. ^13^ for EM images). Next, we determined whether clathrin underlies the pore closure of smaller (~40–150 nm) clathrin-coated pits. Confocal imaging of A655, CLC-mTFP1, and PH-AUX1-mNeonGreen (PHA_G_) that binds and labels both PtdIns(4,5)P_2_ and clathrin^29^, showed that most PHA_G_ puncta co-colocalized with CLC puncta (98 ± 2%, 10 cells; Fig. 4a, b)^29^, which reflected mostly clathrin-coated pits for three reasons. First, 77 ± 3% (10 cells) of PHA_G_/CLC puncta overlapped with A655 spots (Fig. 4a, b), which reflects an A655-containing pit (see Materials and methods for data acquisition and selection that avoided large pre-Ω). Second, most PHA_G_/CLC puncta (93 ±2%, 8 cells) were oval/round-shape (not flat) in the PM-to-cytosol orientation (STED XZ-plane, Fig. 4c), which is consistent with clathrin-coated pits (Fig. 4d). Third, high-resolution STED imaging of SNAP-tagged clathrin or PHA_G_ (see Materials and methods) showed spots or rings with a half-maximum-full-width (Width_h_) of 103 ± 1 nm (303 CLC spot/rings) or 97 ± 2 nm (416 PHA_G_ spot/rings, Fig. 4e); for Width_h_ > 60 nm, 61 ± 7% appeared as rings resembling clathrin-coated pits (Fig. 4e; CLC:112 ± 1 nm, *n* = 194; PHA_G_, 99 ± 3 nm, *n* = 223). This percentage, obtained at single Z-planes, was underestimated, because some spots were rings at different Z-planes (Supplementary Fig. S11).

Depol_1s_ induced PHA_G_/CLC puncta disappearance during confocal XY-plane imaging (14 ± 3%, Fig. 4f–h, 24 cells), which reflected a vesicle pinching off for three reasons. First, while PHA_G_/CLC puncta disappeared, F_655_ decayed without a preceding increase at single (Fig. 4g, left or middle, A655 spot detectable or undetectable) or averaged puncta (Fig. 4h). Thus, PHA_G_/CLC puncta disappearance was not preceded by a flat-to-Ω transition, which would otherwise take up A655 and thus increase F_655_ before F_655_ decays^13^. Second, while PHA_G_/CLC puncta faded, puncta XY-plane movement was observed sometimes (15 ± 2%, 24 cells, Fig. 4g, right). Similarly, XZ-plane imaging showed PHA_G_ puncta disappearance and/or PM-to-cytosol movement (Fig. 4i, *n* = 44 events, 6 cells). These results revealed pinched vesicle movement directly. Third, dynamin inhibitor dynasore blocked the depol_1s_-induced PHA_G_/CLC puncta disappearance (Fig. 4j), suggesting a dynamin-dependent vesicle pinch-off.

Resembling dynasore, si-CHC, or pitstop 2 substantially reduced the depol_1s_-induced PHA_G_/CLC puncta disappearance probability (Fig. 4j), indicating clathrin’s necessity in clathrin-coated vesicle pinch-off. si-CHC also reduced the PHA_G_/CLC puncta number at rest (Supplementary Fig. S12), consistent with clathrin’s potential role in pit formation^4,5^. We concluded that clathrin is required for ~40–150 nm clathrin-coated pit pinch-off (Fig. 4).

### Clathrin mediates slow, fast, ultrafast and overshoot endocytosis by closing pre-Ω/fs-Ω’s pore in chromaffin cells

To recycle exocytosed vesicles, secretory cells use many endocytic modes, including slow (τ > ~ 6 s), fast (τ < ~ 6 s), ultrafast (τ < ~ 0.6 s), and overshoot endocytosis (more endo- than exocytosis). These modes may generate non-clathrin-coated vesicles and are thus considered clathrin (coat)-independent mode of endocytosis^30–32^. Given that clathrin closes Ω-profile’s pore (Figs. 1, 2), here we determined whether these different modes depended on clathrin for pore closure in chromaffin cells containing large (~200–1500 nm), non-clathrin-coated vesicles^15^ and in hippocampal synapses containing small (~30–80 nm) vesicles.

**Fig. 5:**
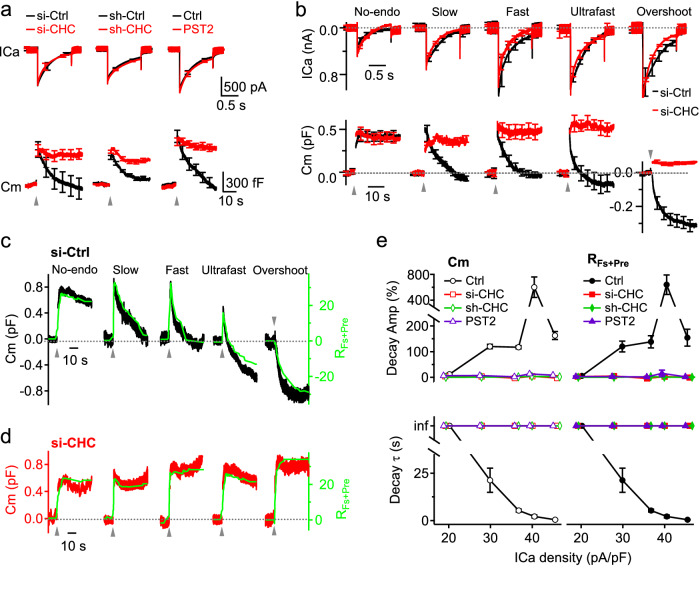
Clathrin-mediated pre-Ω and fs-Ω pore closure underlie slow, fast, ultrafast and overshoot endocytosis in chromaffin cells. **a** ICa and Cm (mean ± s.e.m.) induced by depol_1s_ (gray triangle) in three conditions: (1) si-Ctrl (27 cells) or si-CHC (15 cells); (2) sh-Ctrl (16 cells) or sh-CHC (16 cells); and (3) control (Ctrl, 14 cells) or pitstop 2 (PST2, 30 μM, bath, 15–30 min, 13 cells). **b** Mean ICa and Cm (± s.e.m.) induced by depol_1s_ for Group_no-endo_, Group_slow_, Group_fast_, Group_ultrafast_ and Group_overshoot_ in si-Ctrl (black, *n* = 5–6 cells for each group), and for 5 groups in si-CHC (red, 5–6 cells for each group), which were divided based on ICa density similar to 5 si-Ctrl groups (< 22, 22–32, 32–36, 36–41, > 41 pA/pF). **c** Cm (black, left *Y*-axis) and R_fs+pre_ (green, right *Y*-axis) for 5 individual cells transfected with si-Ctrl showing no-endocytosis, slow, fast, ultrafast or overshoot endocytosis (depol_1s_: gray triangle). R_fs+pre_: the summed number of pre-spot-close and close-fusion (vesicle size corrected, see Methods). **d** Similar to panel (**c**), but for si-CHC (cells selected based on ICa density described in **b**). **e** Left: Cm decay amplitude (Decay Amp, upper) and Cm decay τ (lower) plotted vs ICa density (mean ± s.e.m) for five groups (each group: 3–6 cells) of cells in si-CHC, sh-CHC, pitstop 2 (PST2) or in control (Ctrl_pool_, control data pooled from si-Ctrl, sh-Ctrl and Ctrl). Decay Amp normalized to ΔCm; cell grouping is described in **c**. Right: similar to left but replacing Cm with R_fs+pre_ (cell groups same as in left).

In chromaffin cells, depol_1s_ induced whole-cell capacitance jumps (ΔCm) and decays (Decay_Cm_) reflecting exo- and endocytosis, respectively (Figs. 1a, 5a). The Decay_Cm_ showed 5 endocytic modes in five control-cell groups – no-endocytosis (Group_no-endo_, Decay_Cm_ < 0.3ΔCm), slow (Group_slow_, τ > ~ 6 s), fast (Group_fast_, τ < ~ 6 s), ultrafast (Group_ultrafast_, τ < ~ 0.6 s), and overshoot endocytosis (Group_overshoot_, Decay_Cm_ > 1.3ΔCm), which appeared in this order as ICa density increased (Fig. 5a, b; for more detail, see ref. ^13^). Furthermore, the Decay_Cm_ includes bulk endocytosis detected with imaging as the formation of vesicles larger than the fusing vesicles^13^, resulting in a sudden capacitance decay much larger than a single fusing vesicle’s membrane capacitance^33–35^.

Two sets of data suggest that clathrin-mediated pre-Ω/fs-Ω closure underlies each of these modes, including slow, fast, ultrafast, overshoot, and bulk endocytosis. First, without affecting ICa or ΔCm, si-CHC, sh-CHC or pitstop 2 blocked Decay_Cm_ averaged from all cells (Fig. 5a), or each of the five groups divided based on ICa density, which yielded five endocytic modes in control (Fig. 5b). Second, we reconstructed the exo-endocytosis trace (R_fs+pre_) from individual fusion and pre-close by assigning an up-step at fusion onset and a down-step at close-fusion or pre-close (at F_655_ dimming onset; e.g., Fig. 1c–e; see Materials and methods), and then summing up- and down-steps within a cell. R_fs+pre_ resembled the corresponding cell’s Decay_Cm_ amplitude and time course in five groups (Fig. 5c, d), confirming pre-Ω/fs-Ω closure mediates each detected endocytic mode mentioned above^13^. si-CHC, sh-CHC or pitstop 2 blocked Decay_Cm_ and R_fs+pre_ decay in all groups (Fig. 5d, e), indicating that clathrin underlies each above-mentioned endocytic mode by closing pre-Ω/Fs-Ω.

### Clathrin mediates endocytosis at hippocampal synapses

**Fig. 6:**
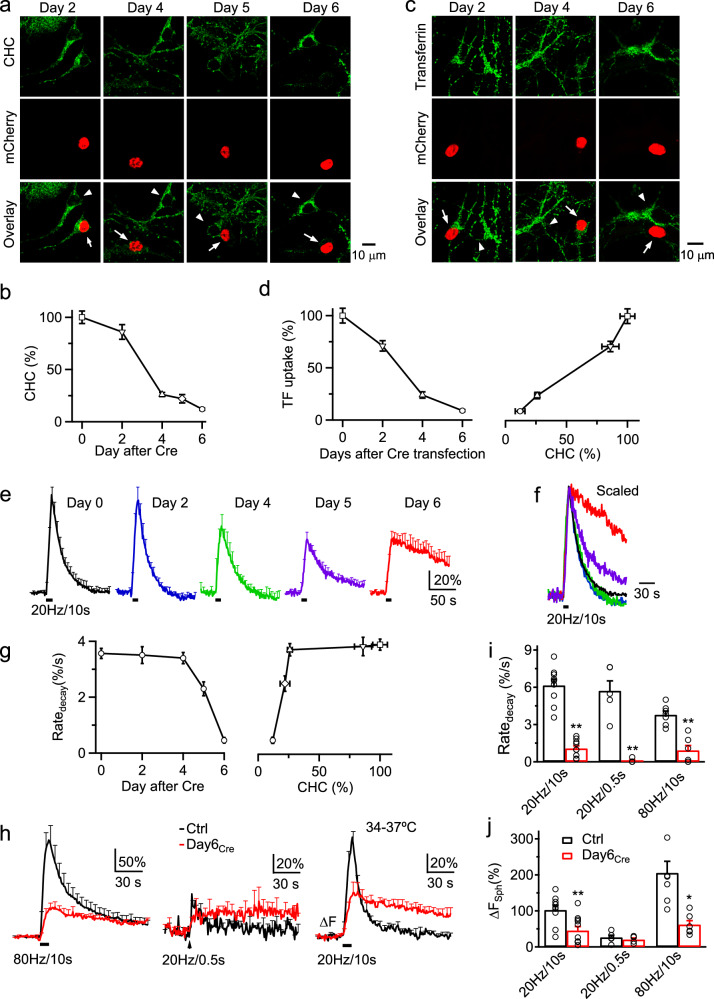
Clathrin is essential for fast and slow endocytosis at hippocampal synapses. **a** CHC antibody staining, and mCherry fluorescence in neurons at 2, 4, 5, and 6 days after Cre/mCherry transfection to *CHC*^*Loxp/Loxp*^ hippocampal culture (confocal images). mCherry fluorescence is shown to distinguish between transfected (arrows) and un-transfected neurons (triangles, control). **b** CHC labelling intensity at Cre/mCherry-transfected neuronal soma at 2–6 days after Cre/mCherry transfection to *CHC*^*Loxp/Loxp*^ culture. Data normalized to the mean of day 0 group taken from un-transfected neurons (each group: 45–65 neurons; 4 transfections, 16 mice). **c** Sampled Alexa 488-conjugated transferrin (TF) uptake in cell bodies of *Chc*^*Loxp/Loxp*^ hippocampal neurons at 2, 4 and 6 days after Cre/mcherry transfection. mCherry fluorescence is shown to distinguish between transfected (arrows) and un-transfected neurons (triangles, control). **d** TF uptake intensity in cell bodies of *Chc*^*Loxp/Loxp*^ hippocampal neurons plotted versus the day after Cre/mcherry transfection (left) or the corresponding CHC level (right, CHC level obtained from **b**). Each data group: 33–59 neurons, 4 transfections, 16 mice. **e** SpH fluorescence trace (F_SpH_, mean ± s.e.m., every 5 s) induced by AP_20H/10s_ in 0 (Ctrl, *n* = 10 experiments), 2 (*n* = 6), 4 (*n* = 5), 5 (*n* = 6), and 6 days (*n* = 10) after Cre/mCherry transfection at 22–24 ^o^C. **f** Traces (s.e.m. not included) in panel e (same color code) re-scaled to the same peak and superimposed for comparison of the decay time course. **g** Rate_decay_ (mean ± s.e.m.) induced by AP_20H/10s_ plotted versus the day after Cre transfection (left) or the corresponding CHC level (right, CHC level obtained from **b**). **h**–**j** F_SpH_ traces (**h**, mean ± s.e.m.), Rate_decay_ (**i**) and F_SpH_ jump (ΔF_SpH_, **j**) induced by AP_20H/10s_, AP_20H/0.5s_ and AP_80H/10s_ in Ctrl (black) and Day6_Cre_ (red) culture at 34–37 ^o^C. Rate_decay_ (**i**, %/s, mean ± s.e.m) was measured from normalized F_SpH_ traces (ΔF_SpH_ was normalized as 100% before Rate_decay_ measurement); ΔF_SpH_ (**h**, mean ± s.e.m) was calculated as the percent increase of F_SpH_ over baseline. Ctrl: *CHC*^*Loxp/Loxp*^ synapses transfected with SpH alone for 6 days. *P < 0.05; **P < 0.01; *t*-test.

To examine synaptic vesicle endocytosis, which is mainly considered clathrin-dispensable^31,32,36–38^ (but see ref. ^39^), we generated CHC conditional knockout (*CHC*^*LoxP/LoxP*^) mice by floxing *CHC* Exon 2 and deleting *CHC* via Cre/mCherry transfection to hippocampal neurons cultured from *CHC*^*LoxP/LoxP*^ mice (Supplementary Fig. S13 and legend). 2–6 days after Cre/mCherry transfection, the protein expression level of CHC (Fig. 6a), but not other proteins, such as AP2, dynamin 1, endophilin 1, SNAP-25 and syntaxin (Supplementary Fig. S14), in neuronal somata expressing mCherry was reduced to ~86%–12% of neighboring un-transfected neurons (Fig. 6b). 2–6 days after Cre/mCherry transfection, transferrin uptake in somata that reflected clathrin-dependent receptor-mediated endocytosis, was reduced to ~71%–9% of control (Fig. 6c, d), confirming that *CHC* gene deletion inhibits clathrin-dependent endocytosis.

A 10-s action potential (AP) train at 20 Hz (AP_20Hz/10s_) induced pH-sensitive synaptophysin-pHluorin 2x (SpH, transfected) fluorescence (F_SpH_) increase (ΔF_SpH_) and decay that reflect exo- and endocytosis, respectively (Fig. 6e)^40,41^. At 5 or 6 days (Day6_Cre_) after Cre transfection, at which CHC was ~22% or ~12% of control (Fig. 6a, b), F_SpH_ decay was prolonged; F_SpH_ decay’s initial rate (Rate_decay_) was inhibited to 65 ± 7% or 13 ± 3% of control (Fig. 6e–g).

Rate_decay_ did not decrease substantially at day 2–4 after Cre transfection at which CHC was ~86%–26% of control, but decreased substantially at day 5–6 at which CHC was ~22%–12% of control (Fig. 6e–g). The relation between Rate_decay_ and CHC level (Fig. 6g, right) was much left-shifted compared to that between transferrin uptake and CHC, as obtained at day 2–6 after Cre transfection (Fig. 6d, right). For example, with ~26% CHC left on day 4, Rate_decay_ was reduced negligibly whereas transferrin uptake was reduced substantially (by ~74%); whereas with ~12% CHC left on day 6, both Rate_decay_ and transferrin uptake were greatly reduced (Fig. 6d, right, 6 g, right). Thus, Rate_decay_ is less sensitive to CHC reduction than receptor-mediated transferrin endocytosis.

Large Rate_decay_ reduction in Day 6_Cre_ cultures was observed regardless of whether the followings: (1) Cre transfection procedure involved lipofectamine (Fig. 6e–g) or calcium-phosphate (Supplementary Fig. S15); (2) SpH (Fig. 6e–g) or synaptobrevin-pHluorin (Supplementary Fig. S16) was transfected; (3) stimulation was AP_20Hz/10s_, 0.5-s AP train at 20 Hz (AP_20Hz/0.5s_) or 10-s AP train at 80 Hz (AP_80Hz/10s_, Fig. 6h, i; Supplementary Fig. S17), or (4) temperature was 22–24 ^o^C (Fig. 6e–g; Supplementary Fig. S17) or 34–37 ^o^C (Fig. 6h, i). Co-transfection of wildtype CHC rescued CHC expression (*n* = 3 transfections) and Rate_decay_ to control (Supplementary Fig. S18). Inhibition of F_SpH_ decay reflected inhibition of surface SpH endocytosis, but not vesicle re-acidification, because the undecayed F_SpH_ was quenched by an acid solution (Supplementary Fig. S19)^42^. These results revealed a robust block of endocytosis in Day6_Cre_ cultures.

In control at 34–37 ^o^C, F_SpH_ decay was bi-exponential after AP_20Hz/10s_ (τ_1_: 7.4 ± 0.5 s, weight: 0.8; τ_2_: 46.0 ± 7.1 s, *n* = 9 experiments), mono-exponential after AP_20Hz/0.5s_ (τ: 12.2 ± 2.1 s, *n* = 5), and bi-exponential after AP_80Hz/10s_ (τ_1_: 13.1 ± 1.3 s, weight: 0.5; τ_2_: 56.0 ± 0.5 s, *n* = 7, Fig. 6h, i). In Day 6_Cre_ cultures, both fast and slow decay components were abolished (Fig. 6h, i), suggesting that clathrin is needed in mediating endocytosis with various speeds. We also observed a ΔF_SpH_ reduction after an AP_20Hz/10s_ and AP_80Hz/10s_, but not AP_20Hz/0.5s_, which depleted the readily releasable pool (Fig. 6j)^30,31,43^; therefore suggesting that clathrin may also facilitate vesicle replenishment to the readily releasable pool^30^.

### Reconciling apparent conflicts of clathrin involvement at synapses

Our results are apparently different from previous studies showing that clathrin knockdown insignificantly affects slow or ultrafast endocytosis^36–38,44^ (but see ref. ^39^) and that pharmacological blockers insignificantly affect fast or ultrafast endocytosis at synapses, particularly hippocampal synapses^45,46^. While these studies led to a current view that clathrin is dispensable for synaptic vesicle endocytosis^31,32^, the possibility that much less clathrin at synapses is required to mediate endocytosis than the classical clathrin-dependent receptor-mediated endocytosis has not been excluded. Supporting this possibility, endocytosis inhibition was only evident when clathrin was reduced by > ~74% (Fig. 6e–g). Reducing clathrin by ~70–75% blocked clathrin-dependent transferrin uptake, but marginally affected synaptic vesicle endocytosis at hippocampal synapses in a study, leading to a suggestion that synaptic vesicle endocytosis is clathrin-dispensable^38^. In contrast, reducing clathrin by ~88% blocked both transferrin uptake and synaptic vesicle endocytosis at hippocampal synapses in another study, leading to a suggestion that clathrin is required for synaptic vesicle endocytosis^39^. These apparently conflicting suggestions can now be reconciled by our finding that the relation between Rate_decay_ and CHC level was much left-shifted compared to that between transferrin uptake and CHC level (Fig. 6g, right, 6d, right). This left-shifted relation indicates that Rate_decay_ is much less sensitive to CHC reduction than transferrin endocytosis, explaining why CHC knockdown, if not sufficient, may block transferrin uptake, but affect marginally the synaptic vesicle endocytosis.

### Both clathrin and dynamin are essential for pore closure

**Fig. 7:**
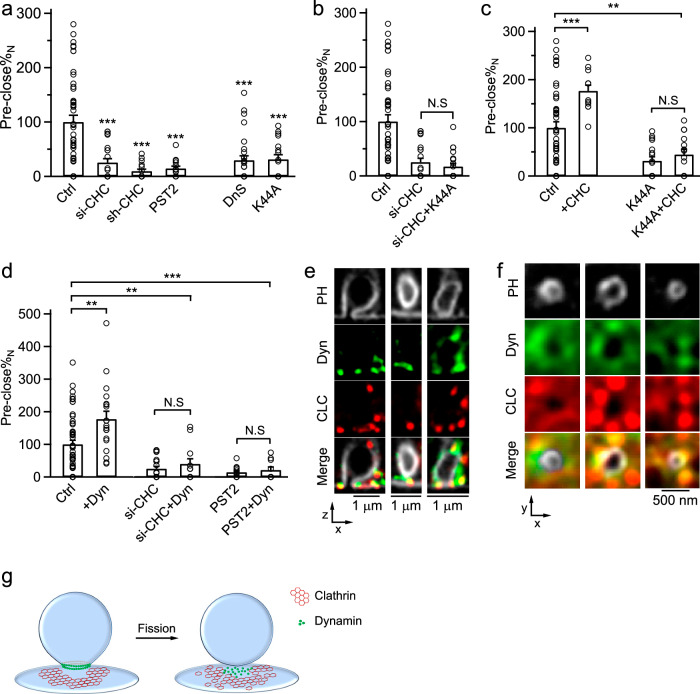
Clathrin or dynamin alone is insufficient to mediate pore closure — both are required to mediate pore closure. **a** Normalized pre-close percentage (Pre-close%_N_) in Ctrl (51 cells), si-CHC (21 cells), sh-CHC (16 cells), pitstop 2 (PST2, 15 cells), dynasore (DnS, 28 cells) or Dyn1-K44A overexpression (K44A, 17 cells). Ctrl data normalized to 100%, other data normalized to Ctrl (applied to **a**–**d**). ***P < 0.001 (compared to Ctrl, *t*-test). **b** Pre-close%_N_ in Ctrl (51 cells), si-CHC (21 cells), or si-CHC plus Dyn1-K44A (si-CHC+K44A, 20 cells). **c** Pre-close%_N_ in Ctrl (51 cells), CHC overexpression (+CHC, 12 cells), K44A (17 cells) or K44A plus CHC (K44A+CHC, 15 cells). **P < 0.01, *t*-test. **d** Pre-close%_N_ in Ctrl (51 cells), dynamin 2 overexpression (+Dyn, 19 cells); si-CHC (21 cells) or si-CHC plus Dyn overexpression (si-CHC+Dyn, 12 cells); PST2 (15 cells) or PST2 plus Dyn overexpression (PST2+Dyn, 10 cells). **e**, **f** STED XZ- (**e**) or XY-plane (**f**) images of PH_G_, CLC (SNAP-CLC-SiR) and Dyn (dynamin 2-mTFP1) at pre-Ω’s pore region. **g** A schematic diagram showing the requirement of both clathrin and dynamin in mediating membrane fission (the post-fission dynamin and clathrin are drawn as disassembled at least partially based on speculation).

Clathrin is crucial for pre-Ω and fs-Ω closure (Fig. 1), clathrin-coated vesicle pinch-off (Fig. 4), and secretory vesicle endocytosis (Figs. 5, 6). Similarly, dynamin is crucial for all these processes shown in many previous studies^5,12,14,47^. On the other hand, filamentous actin, which may generate forces for membrane invagination^48–51^, is not involved in pre-Ω pore closure^9^. Here we present four sets of evidence showing that clathrin or dynamin alone is insufficient to mediate pore closure and that both are required (Fig. 7a–g). First, si-CHC, sh-CHC or pitstop 2 reduced pre-close by ~75–91% (Figs. 1g, 7a). Similarly, dynamin inhibitor dynasore or mutant dynamin 1 K44A overexpression (Dyn1-K44A) reduced pre-close by ~67%–71% (Fig. 7; for detail, see refs. ^9,12–14^). Similarly, a large reduction of close-fusion percentage was observed when clathrin or dynamin was inhibited. The block of most pore closure events by either clathrin or dynamin inhibition (Fig. 7a) suggests that both clathrin and dynamin are required for each single pore-closure event. Second, si-CHC plus Dyn1-K44A inhibited pre-close similar to si-CHC alone (~75%–83%, Fig. 7b). Therefore, Dyn1-K44A could not exert an inhibitory effect when clathrin was knocked down (Fig. 7b), suggesting the requirement of both dynamin and clathrin for pore closure. Third, CHC overexpression in control cells enhanced the pre-close percentage above the control level, whereas CHC overexpression to Dyn1-K44A-transfected cells could not (Fig. 7c), suggesting the requirement of dynamin for clathrin overexpression to enhance pore closure. Likewise, dynamin 2 overexpression in control cells increased the pre-close above the control level, whereas dynamin 2 overexpression in si-CHC-transfected cells or pitstop 2-treated cells could not (Fig. 7d), suggesting the requirement of clathrin for dynamin overexpression to enhance pore closure. Fourth, three-color STED imaging showed that both dynamin and clathrin were associated with the PH_G_-labelled pre-Ω’s pore/base region (145 out of 148 pores), and they may surround the PH_G_-labelled visible or non-visible pore/base region of the Ω-profiles at the XZ- (Fig. 7e) or XY-plane (Fig. 7f). Thus, clathrin and dynamin are physically available around the pore region to mediate pore closure (Fig. 7e, f). We concluded that clathrin and dynamin are both required to provide sufficient constriction forces to close the pore (Fig. 7g).

## Discussion

The present work revealed that clathrin polymerization around the Ω-profile’s base/pore region with an intrinsically curved shape generates elastic forces to constrict and close the pores of ~200–1500 nm pre-Ω and fs-Ω (Figs. 1–3), and ~40–150 nm clathrin-coated pits (Fig. 4). Such a clathrin-dependent pore closure underlies diverse endocytic modes, including ultrafast, fast, slow, overshoot, and bulk endocytosis (Figs. 5, 6), mediates kiss-and-run fusion (Fig. 1), and regulates fusion pore dynamics that determine vesicular content release rates (Fig. 1) in chromaffin cells and hippocampal synapses.

For three reasons, this new function is of a much wider impact than clathrin’s well-known universal function in coating and thus likely in forming Ω-profiles. First, clathrin coating of the Ω-profile’s head is limited to small ~40–150 nm Ω-profiles, whereas clathrin-mediated pore constriction/closure may apply to all Ω-profiles regardless of their sizes (~40–1500 nm) or their head’s coating with clathrin or not (Figs. 1–4). Second, clathrin coating of vesicles is limited to clathrin-coated vesicle endocytosis and budding, whereas clathrin-mediated pore closure may apply not only to clathrin-coated vesicle endocytosis (Fig. 4), but also non-clathrin-coated vesicle endocytosis or budding (Figs. 5, 6). We showed that clathrin mediates all kinetically detectable forms of endocytosis, including ultrafast, fast, slow, bulk, and overshoot endocytosis, in chromaffin cells and synapses (Figs. 5, 6). Since these modes of endocytosis are widely reported in neurons, endocrine cells, and non-secretory cells^30–32,52^, clathrin may mediate fission of these diverse endocytic modes observed in many different cell types. Third, clathrin coats only budding vesicles, whereas clathrin constricts/closes the pore of both budding (pre-Ω) and fusing (fs-Ω) vesicles (Fig. 1). Consequently, clathrin is essential to mediating fusion pore closure (Fig. 1), the widely reported kiss-and-run fusion previously considered the ‘bona-fide’ clathrin-independent fusion^12,17,30,53–55^. By constricting the fusion pore, clathrin counteracts fusion pore expansion and thus inhibits vesicular content release (Fig. 1). Clathrin may therefore control important secretion-related functions previously unrecognized, such as synaptic transmission, fight or flight responses, immune responses, and regulation of diabetes-relevant blood glucose levels^30,56^. In brief, the traditional function of clathrin in coating an Ω-profile’s head is limited to small budding vesicles, whereas clathrin’s pore-constriction/closure function may apply widely to all different sizes of budding and fusing vesicles coated with or without clathrin to control vesicle endocytosis, intracellular trafficking, and exocytosis.

Clathrin coating of vesicles has been used to classify vesicle endocytosis and budding into clathrin-dependent and -independent modes^5,6,57^. Our results suggest a thorough redefining of this half-a-century-old concept, because all clathrin (coat)-independent modes of endocytosis previously classified in secretory cells, such as ultrafast^36,37^, fast^45^, slow^38^, bulk, and overshoot endocytosis, as well as kiss-and-run^30,53^, depend on clathrin for pore closure as demonstrated here (Figs. 1–5). We suggest redefining these endocytic modes and kiss-and-run fusion as the clathrin-dependent mode regarding the pore closure. Numerous previous studies interpreting results based on the old concept of the clathrin (coat)-dependent endocytosis or budding may need to be re-examined. The widely held view that clathrin is indispensable for synaptic vesicle endocytosis^36–38,44^ (but see ref. ^39^) is likely due to insufficient clathrin knockdown or inhibition, as shown here at hippocampal synapses (Fig. 6).

We found that clathrin generates constriction forces by polymerization at the base/pore region, explaining how clathrin constricts and closes the pore of non-clathrin-coated Ω-profiles (referring to no clathrin coating at the Ω-profile’s head; Figs. 1–3). This mechanism may also explain clathrin-coated Ω-profiles’ pore closure (Fig. 4), if clathrin also polymerizes at the base/pore region of clathrin-coated Ω-profiles. Alternatively, polymerized clathrin at the head of the Ω-profile may also generate elastic forces to constrict the clathrin-coated Ω-profile’s pore, as suggested by an early mathematical modelling study^26^. Taken together, clathrin polymerization at the Ω-profile’s base/pore and/or head may generate elastic forces to constrict the Ω-profile’s pore, mediating fission of non-clathrin-coated or clathrin-coated vesicles. Clathrin is thus an essential component of the fission machinery.

The extent of clathrin polymerization at the Ω-profile’s base/pore region might contribute to explaining the speed of endocytosis. If clathrin is largely polymerized, further polymerization may take much less time and thus contribute to mediating ultrafast or fast endocytosis. On the contrary, if clathrin is much less polymerized, further polymerization towards completion may take a longer time and thus contribute to mediating slow endocytosis. It is unclear how clathrin is recruited to the Ω-profile’s base/pore region. Many mechanisms may contribute to recruiting clathrin, such as the random stochastic collision of endocytic adaptor proteins^58^, the involvement of PI(4,5)P_2_ in recruiting adaptors that may bind clathrin, the endocytic cargos that recruit clathrin adaptors to bind clathrin^59,60^, and the curvature that may recruit adaptors and clathrin^61^ (see also review in refs. ^4,5^). It would be of interest to study how clathrin is recruited to the Ω-profile’s base/pore region in the future.

Since the intrinsically curved shape of polymerized clathrin produces pore-constriction forces (Fig. 3), this mechanism may, in principle, apply to other membrane coat-forming proteins with an intrinsically curved shape, such as COP I/II and caveolin^2,5,11,27^. Such a mechanism may explain dynamin-independent vesicle budding, including vesicle endocytosis reported in many cell types like neurons, yeast, pancreatic β-cells, and fibroblasts^62–66^.

While clathrin is crucial for pore closure of pre-Ω, fs-Ω, clathrin-coated pits and synaptic vesicles (Figs. 1–6), dynamin is also essential for each of these pore-closure events (Figs. 4, 7)^12–14,34,62,67–72^. This finding suggests that neither dynamin nor clathrin alone, but the two together generate constriction forces sufficient to close the Ω-profile’s pore in live cells, challenging the long-held concept that dynamin alone provides forces driving fission^11,27^. We suggest modifying this concept to include both dynamin and clathrin in providing constriction forces essential for fission, but with two different mechanisms: the pore-surrounding dynamin helix constriction together with constriction forces generated by clathrin polymerization at the Ω-profile’s base/pore and/or head region. This modification may be of widespread application, given that both dynamin and clathrin are ubiquitous proteins involved in intracellular trafficking, endocytosis, membrane-bound organelle generation and exocytosis. Although dynamin alone can mediate fission in vitro^11,27^, in vivo protein and lipid composition, membrane tension, and pore geometry may differ from in vitro conditions, which may explain the need for more forces cooperating to constrict and close the Ω-profile’s pore in live cells.

In summary, clathrin polymerization into an intrinsically curved coat produces elastic forces to constrict and close the fission or fusion pores of ~40–1500 nm non-clathrin-coated or clathrin-coated Ω-profiles. This mechanism may underlie and/or regulate diverse endocytic modes, kiss-and-run fusion, vesicular content release, intracellular trafficking, membrane-bound organelle formation, and viral infection in most cells. Therefore, clathrin plays much broader and more important roles than previously recognized in coating small budding vesicles, calling for a thorough redefining of the half-a-century-old concept regarding clathrin-dependent and -independent endocytosis or budding. Clathrin is an essential component of the fission machinery in live cells, calling for modifying the fission-machinery concept, which contains only the helix-forming proteins as force-generators, to include membrane protein coats with an intrinsic curvature that may generate pore-constriction/closure forces.

## Materials and methods

### Chromaffin cell culture

We prepared primary bovine adrenal chromaffin cell culture as described previously^14,18,73,74^. Fresh adult (21–27 months old) bovine adrenal glands (from a local abattoir) were immersed in pre-chilled Locke’s buffer on ice containing: 145 mM NaCl; 5.4 mM KCl; 2.2 mM Na_2_HPO_4_; 0.9 mM NaH_2_PO_4_; 5.6 mM glucose; 10 mM HEPES (pH 7.3, adjusted with NaOH). Glands were perfused with Locke’s buffer, then infused with Locke’s buffer containing collagenase P (1.5 mg/mL, Roche), trypsin inhibitor (0.325 mg/mL, Sigma), and bovine serum albumin (5 mg/mL, Sigma), and incubated at 37 °C for 20 min. The digested medulla was minced in Locke’s buffer, and filtered through a 100-μm nylon mesh. The filtrate was centrifuged (48× *g*, 5 min), re-suspended in Locke’s buffer and re-centrifuged until the supernatant was clear. The final cell pellet was re-suspended in pre-warmed DMEM medium (Gibco) supplemented with 10% fetal bovine serum (Gibco).

### Electroporation and plating for chromaffin cell culture

Cells were transfected by electroporation using Basic Primary Neurons Nucleofector Kit (Lonza), according to the manufacturer’s protocol and plated onto glass coverslips with mouse Laminin coating over PDL layer (Neuvitro)^73,74^. The cells were incubated at 37 °C with 9% CO_2_ and used within 5 days.

### Plasmids, siRNA, shRNA, fluorescent dyes and blockers used for chromaffin cells

The PH-EGFP (phospholipase C delta PH domain attached with EGFP) was obtained from Dr. Tamas Balla. PH-mCherry, PH-mTFP1, PH-mScarlet and PH-mNeonGreen (PH_G_) construct were created by replacing the EGFP tag of PH-EGFP with mCherry, mTFP1, mScarlet, and mNeonGreen (Allele Biotechnology)^75^, respectively. CLC-mTFP1 and CLC-mNeonGreen construct were created by replacing the EGFP tag of CLC-EGFP (gift from Dr. Lois Greene) with mTFP1 (Addgene# 54521) or mNeonGreen (Allele Biotechnology). SNAP-CLC construct was created by replacing the EGFP tag of CLC-EGFP with SNAP (Addgene# 58187). The EGFP tag of Aux1-based PtdIns(4,5)P_2_ sensor EGFP-PH-Aux1 (PHA-EGFP, gift from Dr. Tom Kirchhausen) was replaced with mNeonGreen or SNAP to create PHA_G_ and SNAP-PHA. Dynamin 2-mTFP1 and dynamin 2-HALO were generated from pEGFP-N1-dynamin 2 plasmid (Addgene# 34686) by replacing EGFP with mTFP1 and HALO tag, respectively. Dynamin 1-K44A-EGFP and dynamin 1-K44A-mCherry was generated from dynamin 1-K44A-mRFP (Addgene# 55795) by replacing mRFP with EGFP and mCherry, respectively.

For knockdown of endogenous CHC in bovine chromaffin cells, three siRNA duplexes for bovine *CHC* (5′-CCAGAUUGUCGAUGUGUUU-3′, 5′-GCAUCUACAUGAAUAGAAU-3′, and 5′-CUAUGACAGUCGUGUUGUU-3′) labeled with Cyanine 3 and scrambled control siRNA (SIC003) were purchased from Sigma-Aldrich. A mixture of these three siRNAs was used for experiments. shRNA plasmids for bovine *CHC* (5′-CGGTTGCTCTTGTTACGGATAATGCAGTT-3′) and scrambled control (TR30015) tagged with RFP were purchased from Origene. The C1573S CHC mutant was generated from pEGFP-C1-CHC17(Addgene# 59799) plasmid.

When Atto 532 (A532, Sigma), Atto 655 (A655, Sigma) or Alexa 488 (A488, Sigma) was included in the bath solution, the dye concentration was 30 μM. For FFN511 (Abcam) imaging, cells were bathed with FFN511 (5–10 μM) for 20 min and images were performed after washing out FFN511 in the bath solution. Pitstop 2 (30 μM, bath, 15–30 min) and dynasore (80 μM, bath, 20–30 min) were purchased from Millipore Sigma.

### Western blot analysis

Total protein was extracted from cultured chromaffin cells using RIPA buffer containing protease inhibitor cocktail (Millipore Sigma). Equal amounts of proteins, determined by BCA protein assay (Invitrogen) were loaded onto 4%–12% Bis-Tris gel (Invitrogen). Proteins were transferred onto PVDF membrane and immunoblotted with indicated primary antibodies at 4 ^o^C overnight. Membranes were incubated with HPR labeled secondary antibodies and visualized using Bio-Rad ChemiDoc Imaging System. Primary antibodies including mouse anti-CHC (1:500, Abcam), rabbit anti-dynamin (1:1000, Cell Signaling Technology), mouse anti-AP2 (1:1000, ThermoFisher Scientific), anti-Synaptotagmin 1 (1:500, Synaptic Systems), mouse anti-Syntaxin (1:500, Abcam) and β-actin (1:3000; Abcam).

### Electrophysiology for chromaffin cells

At room temperature (20–22 °C), whole-cell voltage-clamp and capacitance recordings were performed with an EPC-10 amplifier together with the software lock-in amplifier (PULSE, HEKA, Lambrecht, Germany)^18,73,74,76^. The holding potential was –80 mV. For capacitance measurements, the frequency of the sinusoidal stimulus was 1000–1500 Hz with a peak-to-peak voltage ≤ 50 mV. The bath solution contained 125 mM NaCl, 10 mM glucose, 10 mM HEPES, 5 mM CaCl_2_, 1 mM MgCl_2_, 4.5 mM KCl, and 20 mM TEA, pH 7.3 adjusted with NaOH. The pipette (2–4 MΩ) solution contained 130 mM Cs-glutamate, 0.5 mM Cs-EGTA, 12 mM NaCl, 30 mM HEPES, 1 mM MgCl_2_, 2 mM ATP, and 0.5 mM GTP, pH 7.2 adjusted with CsOH. These solutions pharmacologically isolated calcium currents.

For stimulation, we used a 1-s depolarization from the holding potential of –80 mV to +10 mV (depol_1s_). We used this stimulus, because it induces robust exo-endocytosis as reflected in capacitance recordings (Fig. 1a)^18,77,78^.

### STED imaging for chromaffin cells

We performed STED imaging as described previously^12,13,73^. If not mentioned, STED images were acquired with Leica TCS SP8 STED 3× microscope that is equipped with a 100× 1.4 NA HC PL APO CS2 oil immersion objective and operated with the LAS-X imaging software. Excitation was with a tunable white light laser and emission was detected with hybrid (HyD) detectors. PH_G_ and A532 were sequentially excited at 485 and 540 nm, respectively, with the 592 nm STED depletion beam, and their fluorescence collected at 490–530 nm and 545–587 nm, respectively.

The excitation power for A532 was 10% of the maximum, at which fluorescent A532 can be bleached within a few seconds during repeated XZ-plane imaging (Y-plane fixed) every 26–200 ms. This feature was used to distinguish whether the PH_G_-labelled Ω-profile’s pore is closed or not, because pore closure prevents bleached A532 (caused by strong excitation) from exchange with fluorescent A532 in the bath, resulting in A532 spot fluorescence decay^12,18^. In contrast, an open pore would not cause A532 spot fluorescence decay, because an open pore allows for continuous exchange of bleached A532 in the Ω-profile with fluorescent A532 in the bath^12,18^.

For three-color STED imaging with 592 nm STED depletion laser, PH-mNeonGreen, CLC-mTFP1, and A532 were excited at 488 nm, 442 nm, and 540 nm, respectively, and their fluorescence collected at 493–535 nm, 447–482 nm, and 545–587 nm, respectively.

For STED at the XZ-plane with a fixed *Y*-axis location (e.g., Fig. 2d, e, g), images were acquired at XZ-plane every 26–200 ms at 15 nm per pixel in an XZ area of 15–20 μm × 0.7–2.5 μm, with a fixed *Y*-axis location at about the cell center (Fig. 1a). The imaging duration was limited to 10–20 s before, and 60 s after depol_1s_. We limited to 60 s, because whole-cell endocytosis after depol_1s_, measured with capacitance recordings, usually takes place within 60 s (e.g., Fig. 1a)^12,14,18^. Each cell was subjected to only 1 depol_1s_ to avoid endocytosis rundown^79^. For these time-lapse experiments, we did not scan images in multiple *Y*-axis locations to obtain a volume scanning, because such a volume scanning may significantly reduce the time resolution and substantially bleach fluorophores.

The STED resolution for imaging PH_G_ in our conditions was ~60 nm on the microscopic *X*- and *Y*-axis (parallel to cell-bottom membrane or coverslip), and ~150–200 nm on the microscopic *Z*-axis. As described previously^73^, STED images were deconvolved using Huygens software (Scientific Volume Imaging) and analyzed with Image J and LAS X (Leica).

Some STED images were acquired from Abberior Expert Line system based on inverted Olympus IX83 microscope equipped with an UPlanSApo 100× 1.4 NA oil immersion objective and high power 775 nm STED depletion laser that may provide a higher STED resolution. Chromaffin cells were transfected with (1) SNAP-PH-AUX1 (SNAP-PHA), (2) SNAP-CLC, (3) SNAP-CLC and PH_G_ (2-color STED), or (4) SNAP-CLC, Dyn2-HALO and PH_G_ (3-color STED). Cells were incubated for 30 min. with 0.3 µM SNAP-Cell® 647-SiR (NEB, 8102 S) and/or 1 µM JF585 HaloTag® ligand (Promega, CS315105) for the attachment of SNAP-Cell® 647-SiR to SNAP-PHA (SNAP-PHA-SiR) or SNAP-CLC (SNAP-CLC-SiR), and for the attachment of JF585 to Dyn2-HALO (Dyn2-HALO-JF585).

For one-color STED imaging of SNAP-PHA-SiR or SNAP-CLC-SiR (Fig. 4e), excitation (ex) was with 640 nm laser; STED depletion was conducted with 775 nm depletion beam; emission fluorescence (em) was collected at 650–754 nm. The excitation power for 647-SiR was 1%–5% of the maximum (maximum power: 1 mW) and the 775 nm depletion laser power was set at 5%–10% of the maximum (maximum power: 3 W).

For two-color STED imaging (Fig. 2b), we first acquired SNAP-CLC-SiR images with 775 nm depletion laser (see detailed parameters described above), and then PH_G_ images with 595 nm depletion laser (595 nm depletion laser: 5%–10% maximum power (maximum power: 400 mW); ex: 512 nm laser, 1%–5% maximum power (maximum power: 0.3 mW); em: 525–575 nm).

Three-color imaging of SNAP-CLC-SiR, Dyn2-HALO-JF585 and PH_G_ (Fig. 7e, f) was performed similarly to the two-color imaging as described above, except that Dyn2-HALO-JF585 also was imaged with 775 nm depletion laser. SNAP-CLC-SiR: 640 nm (ex), 650–754 nm (em); Dyn2-HALO-JF585: 561 nm (ex), 571–630 nm (em); PH_G_: 512 nm (ex), 525–551 nm (em).

### Detection of pore closure with STED imaging

During repeated XZ-plane imaging (Y-plane fixed), A532 was excited at a high laser power so that fluorescent A532 can be bleached with a time constant of 1.5–3.5 s. Pore closure was identified as the gradual dimming of the A532 spot fluorescence to baseline during repeated XZ-plane PH_G_/A532 imaging. A532 fluorescence dimming is due to pore closure that prevents bleached A532 (by strong excitation) from exchange with a large reservoir of fluorescent A532 (very small molecule,~1.2 nm) in the bath. This is not due to a narrow pore smaller than A532 molecule size, because after spot dimming, bath application of an acid solution cannot quench the pH-sensitive VAMP2-EGFP or VAMP2-pHluorin overexpressed at the same spot, indicating that the spot is impermeable to H^+^ or OH^–^, the smallest molecules, and thus is closed^14,18^. Furthermore, the closure time course calculated from spot dimming matches approximately with whole-cell endocytosis time course^18^, and inhibition of dynamin by dynamin inhibitors, dynamin dominant-negative mutant dynamin 1-K44A, or dynamin knockdown blocks not only whole-cell endocytosis but also pore closure detected with the spot dimming method^14,18^. These results further confirm that spot dimming under strong excitation reflects pore closure.

### Confocal imaging at chromaffin cells

Confocal imaging of pore closure has been described in detail previously^13,73,74^. Imaging of PH_G_, A655 and FFN511 was performed with an inverted confocal microscope (TCS SP5II, Leica, Germany, 100× oil objective, numerical aperture: 1.4). PH_G_ was excited by a tunable white light laser at 515 nm (laser power set at ~1–4 mW); A655 was excited by an HeNe laser at 633 nm (laser power set at ~12–15 mW); FFN511 was excited by an Argon laser at 458 nm (laser power set at ~2–4 mW); their fluorescence was collected at 520–600 nm, 650–800 nm, and 465–510 nm, respectively. Confocal imaging area was ~70–160 μm^2^ at the XY plane with a fixed *Z*-axis focal plane ~100–200 nm above the cell-bottom membrane. Images were collected every 40–80 ms at 40–60 nm per pixel.

Confocal XY-plane imaging of PHA_G_, CLC-mTFP1 and A655 (Fig. 4a) were performed with prolonged averaging (~64–128 frames) to resolve A655 spots that may fill a tiny Ω-profile. These small spots were not visible in experiments performed for data shown in Figs. 1 and 2 (see Materials and Methods for more detail). PHA_G_/CLC-mTFP1/A655 spots with a half-maximum-full-width more than 300 nm were not selected, which avoid contamination with large pre-Ω analysed in Fig. 1.

During confocal imaging of PH_G_ (weak excitation), A655 (strong excitation) and FFN511 (weak excitation) at the cell-bottom, fusion was detected as FFN511 fluorescence (F_FFN_) decrease with PH_G_ fluorescence (F_PH_) and A655 fluorescence (F_655_) increase. These changes reflect FFN511 release and PH_G_/A655 spot formation by PH_G_/A655 diffusion from plasma membrane/bath into fusion-generated Ω-profiles^12,13,19^. As previously characterized^12,18,19^, fusion could be: (1) close-fusion, detected as F_655_ dimming while F_PH_ sustained or decayed with a delay (Fig. 1d); (2) stay-fusion, the persistent PH_G_/A655 spot presence (Fig. 1e); or (3) shrink-fusion, the PH_G_/A655 spot-size decrease with approximately parallel decay of F_655_ and F_PH_ (Fig. 1e).

### Fusion modes and pre-close detection with confocal microscopy

Fusion-generated Ω may close its pore at ~0.05–30 s later (close-fusion, Fig. 1d), maintain an open pore (stay-fusion, Fig. 1e), or shrink to merge with the plasma membrane (shrink-fusion, Fig. 1e)^12,19^. Close-fusion was detected as A655 fluorescence (F_655_, strongly excited) dimming due to pore closure that prevented bath fluorescent A655 from exchanging with bleached A655, while F_PH_ (weakly excited) sustained or decayed with a delay that reflected PtdIns(4,5)P_2_ conversion into PtdIns(4)P and/or vesicle pinch off (Fig. 1d); stay-fusion was detected as sustained F_655_ and F_PH_; shrink-fusion, A655 and PH_G_ spot shrinking with parallel decreases of F_655_ and F_PH_ (Fig. 1e)^12,14,19^. Pre-close was detected as A655 fluorescence (F_655_, strongly excited) dimming due to pore closure that prevented bath fluorescent A655 from exchanging with bleached A655, while F_PH_ (weakly excited) sustained or decayed with a delay (Fig. 1c).

Pore closure (close-fusion and pre-close) detected with spot F_655_ bleaching by strong excitation is not due to a narrow pore smaller than A655 molecule size, because after spot dimming, bath application of an acid solution cannot quench the pH-sensitive VAMP2-EGFP or VAMP2-pHluorin overexpressed at the same spot, indicating that the spot is impermeable to H^+^ or OH^–^, the smallest molecules, and thus is closed^13,14,18^. Furthermore, pore closure detected with this method was blocked by dynamin inhibitor dynasore, dynamin dominant-negative mutant dynamin 1-K44A, or dynamin knockdown, suggesting that fusion pore closure is mediated by dynamin^13,14,18^.

For imaging of A655 and CLC-EGFP or imaging of A655 and A488, excitation wavelength was 640 nm and 488 nm, respectively; fluorescence emission was collected at 650–800 nm and 495–600 nm, respectively. Without FFN511, fusion was identified as the sudden appearance of A655 spot, the fluorescence of which reached the peak within 20–200 ms. This method was verified with concurrent confocal or STED imaging of NPY-EGFP release or FFN511 release, and with STED imaging of the sudden appearance of (within single frame: ~26–200 ms) of PH_G_-labelled Ω containing A532 spot or releasing FFN511^12,18,19^.

### Reconstruction of exo-endocytosis from individual vesicle fusion, close-fusion and pre-close

To reconstruct the exo-endocytosis trace from individual fusion, close-fusion, and pre-close events (R_fs+pre_), we assigned a 1-unit up-step at each fusion’s onset, a 1-unit down-step for each close-fusion’s onset (at F_655_ dimming onset, e.g., Fig. 1d, e), and a down-step at each pre-close’s onset (A655 bleaching onset; e.g., Fig. 1c). The down-step amplitude of the pre-close was calculated as

$${{\rm{down}}{-}{\rm{step}}\ {\rm{size}}=({\rm{mean}}\ {\rm{pre}}{-}{\rm{close}}\ {\rm{spot}}\ {\rm{width}}/{\rm{mean}}\ {\rm{fusion}}\ {\rm{spot}}\ {\rm{width}})}^{2}$$

where pre-close spots and fusion spots were taken from the same cell (down-step size range: 1.2–2.8, 27 cells). This amplitude rescaling was necessary because the pre-spot size (half width) was on average about 1.38 times as large as the fusion spot^13^. Summing all up and down-steps in each cell yielded the reconstructed R_fs+pre_, which reflects vesicle fusion, close-fusion, and pre-close observed at the cell bottom (e.g., Fig. 5c–e).

### MINFLUX nanoscopy

#### Cell preparation and SNAP labeling

Chromaffin cells transfected with PH-mScarlet and SNAP-CLC were fixed with paraformaldehyde (PFA, 2.4%) and sucrose (2.4%) solution for 30 min. Excess PFA was quenched with 50 mM NH_4_Cl solution for 10 min. and incubated with Image-iT Signal Enhancer solution for 30 min. at room temperature. Subsequently, cells were incubated at room temperature for 50 min with SNAP substrate dye solution containing 1 µM SNAP-Surface^®^ Alexa Fluor^®^ 647 (NEB, S9136S), 0.5% bovine serum albumin, and 1 mM DTT, resulting in the attachment of SNAP-surface® Alexa fluor 647 to SNAP-CLC (SNAP-CLC-A647), which was used for MINFLUX imaging. Next, an undiluted dispersion of gold beads (EM.GC150/4, BBI Solutions) was incubated for 10 min and rinsed of several times with PBS to remove unbound gold beads. The coverslips containing chromaffin cells were mounted on a depression slide with the MINFLUX imaging buffer containing 50 mM Tris/HCl, 10 mM NaCl, 10% (w/v) glucose, 64 µg/mL catalase, 0.4 mg/mL glucose oxidase, and 15 mM MEA at pH 8.0. The coverslips were sealed with Elite double 22 dental epoxy (Zhermack).

#### MINFLUX data acquisition

MINFLUX imaging was performed on a commercial 3D MINFLUX microscope that was driven by the Imspector software with MINFLUX drivers (Abberior Instruments)^80^. Generally, fields of view with multiple gold beads were chosen and locked in a 3D set position for active sample stabilization using the near-infrared scattering from gold beads and active feedback correction via the piezo stage. It was ensured that the standard deviation of the sample position relative to the stabilization set point was less than 2 nm in all directions during measurements. Fields of view were chosen close to the coverslip surface at the bottom of the cell expressing both fusion proteins. Before starting the MINFLUX data acquisition, the fluorophores were driven into the dark state using iterative confocal scans with the 640 nm excitation laser and a power between 8%–15% (maximum power at periscope: 1.94 mW). The sample was imaged with the standard MINFLUX imaging sequence provided by the manufacturer using 10% fixed laser power. During the MINFLUX measurement, the 405 nm activation laser power was ramped up slowly from 0% to 50% over several hours (maximum power at periscope: 27 μW). Samples were generally imaged for 12–24 h.

#### MINFLUX data analysis

The raw final valid molecule position estimates were exported directly from the MINFLUX Imspector interface as a .mat file. Custom MATLAB analysis software was then used to identify and segregate clusters of localizations. The data were filtered to remove traces (group of localizations originating from the same fluorophore emission burst) with a standard deviation of more than 10 nm and less than three localizations per trace^81,82^.

### Platinum replica transmission electron microscopy

Chromaffin cells were rinsed in intracellular buffer (70 mM KCl, 30 mM HEPES maintained at pH 7.4 with KOH, 5 mM MgCl_2_, 3 mM EGTA), and manually unroofed with a 19-gauge needle and syringe using 2% paraformaldehyde (Electron Microscopy Sciences, 15710) in the intracellular buffer^7^. After unroofing, the coverslips were transferred to 2% glutaraldehyde (Electron Microscopy Sciences, 16019) at 4 °C until EM sample preparation. For correlative analysis, they were transferred to fresh 2% paraformaldehyde in the intracellular buffer for 20 min. They were washed with phosphate-buffered saline (PBS). Unroofed cells were stained with ~50 nM of Alexa Fluor 647-phalloidin (Life Technologies, A22287) for 15 min. Cells were then rinsed with PBS. 1 mm × 1 mm fluorescent montage images were generated for EGFP and Alexa Fluor 647 using a Nikon Eclipse Ti inverted microscope with a 100× 1.49 NA objective (Nikon, SR HP Apo TIRF) and an Andor iXon Ultra 897 EM-CCD camera under the control of Nikon Elements software. This spatial map was used to locate transfected cells^83^. The imaged area was marked with a circle (4 mm in diameter) around the center of the imaged area using an objective diamond scriber (Leica, 11505059). The immersion oil was carefully removed from the bottom of the glass coverslip. The sample was stored in 2% glutaraldehyde at 4 °C until EM sample preparation.

EM samples were prepared as described previously^84^. Briefly, coverslips were transferred from glutaraldehyde into 0.1% w/v tannic acid for 20 min. They were rinsed 4 times with water and placed in 0.1% w/v uranyl acetate for 20 min. The coverslips were dehydrated, critical point dried with a critical point dryer (Tousimis Samdri, 795), and coated with platinum and carbon with a freeze fracture coating device (Leica, EM ACE 900). The region of interest on the coverslip marked by a diamond scriber was imaged in brightfield with a 20× phase-contrast objective to obtain another map of the region imaged in fluorescence. The replicas were lifted and placed onto formvar/carbon-coated 75-mesh copper TEM grids (Ted Pella, 01802-F) that were freshly glow-discharged with a PELCO easiGlow 91000. Again, the grid was imaged in brightfield light with a 20× phase-contrast objective to find the same region that was originally imaged in fluorescence. Each cell of interest was located on the grid prior to EM imaging. TEM imaging was performed on a JEOL 1400 equipped with a CMOS camera (NanoSprint43 Mk-II, AMT) at 10,000× or 6000× magnification (0.71 or 1.19 nm per pixel) using SerialEM freeware for montaging^85^. Stitched electron microscopy montages were assembled using IMOD freeware^86^.

Identification of the apparently tilted membrane invagination is described in the main text and in Supplementary Fig. S9 and its legends.

### *CHC*^*Loxp/Loxp*^ mouse, culture and imaging

*CHC*^*Loxp/Loxp*^ mouse generation is described in Supplementary Fig. S13 (genotypes determined by PCR). Breeding *CHC*^*LoxP/LoxP*^ mice with actin β-Cre or synapsin-Cre mice that express Cre broadly produced no *CHC* knockout mice (> 30 pups from > 5 litters), likely due to embryonic death.

Hippocampal CA1-CA3 regions from P0 wildtype or *CHC*^*Loxp/Loxp*^ mice were dissected, dissociated, and plated on Poly-D-lysine. Cells were maintained at 37 °C in a 5% CO_2_ humidified incubator in a medium containing MEM, 0.5% glucose, 0.1 g/L bovine transferrin, 0.3 g/L glutamine, 10% fetal bovine serum, 2% B-27, and 3 µM cytosine β-D-arabinofuranoside. On 6–8 days after plating, neurons were transfected with plasmids using Lipofectamine LTX. Neurons were then maintained at 37 °C for 4–8 days before imaging.

Cultures were transfected with a plasmid containing SpH (or Synaptobrevin-pHluorin, gift from Dr. Yongling Zhu) alone (control) or with L309 plasmid containing Cre/mCherry. A nuclear localization sequence was tagged at the N-terminal of Cre, and cloned into L309 vector via *BamH*I and *EcoR*I sites. Accordingly, mCherry was expressed in the nucleus (Fig. 6a). For CHC rescue, we transected a plasmid (pmCherry-C1-CHC17) containing wildtype CHC and mCherry. pmcherry-C1-CHC17 was generated from pEGFP-C1-CHC17 plasmid by replacing EGFP with mCherry. Without the nuclear localization sequence in the pmCherry-Cl-CHC17 plasmid, mCherry expression was not limited to nucleus (Supplementary Fig. S18a).

Action potential was evoked by a 1 ms pulse (20 mA) through a platinum electrode. The bath solution contained (in mM): 119 NaCl, 2.5 KCl, 2 CaCl_2_, 2 MgCl_2_, 25 HEPES (pH 7.4), 30 glucose, 0.01 6-cyano-7-nitroquinoxaline-2, 3-dione (CNQX), and 0.05 D, L-2-amino-5-phosphonovaleric acid.

For acid quenching, we replaced HEPES in the bath with MES-buffered (25 mM) solution (pH 5.5). For transferrin uptake, cells were incubated with serum-free MEM for 30 min at 37 °C, and then kept in serum-free MEM containing 50 μg/mL Alexa 488 conjugated transferrin for 20 min at 37 °C. Cells were washed twice in 1× PBS and then fixed with 4% paraformaldehyde before mounting for imaging (Leica SP8 confocal microscope).

Except mentioned, SpH (pHluorin) images were acquired at 1–2 Hz with Leica SP8 confocal microscope (Objective: 63×, 1.4 NA). Varicosities (2 × 2 μm) responded to stimulation were analyzed with Leica software. Rate_decay_ after a train of APs at 20–80 Hz was measured as the decay rate in the first 4–10 s after fluorescence increase. Before calculating Rate_decay_, F_SpH_ trace was normalized with ΔF = 100%. Thus, Rate_decay_ reflects the initial decay of F_SpH_ in the percentage of ΔF per second. Each data group was obtained from ≥ 3 cultures. For each experiment, 10–30 boutons were used.

To study endocytosis after an AP, we induced an AP at 0.03–0.05 Hz, which caused a detectable ΔF at a probability of 0.15 per bouton in control (9 experiments, 73 boutons), consistent with a previous report (21). F_SpH_ increase after an AP was identified if it was > 3 times baseline F_SpH_ s.d.

### Immunohistochemistry

Cells were fixed with 4% paraformaldehyde, permeabilized with 0.3% Triton X-100, and subsequently incubated with primary and secondary antibodies. Primary antibodies were diluted in PBS containing 10% donkey serum and incubated with cells for 1 h at 22–24 ^o^C. After several rinses in PBS, cells were incubated with fluorescence-conjugated donkey anti-mouse, anti-sheep, or anti-rabbit IgG (1:1000, Invitrogen) for 2 h at 22–24 ^o^C. Primary antibodies included mouse anti-CHC (1:500, Abcam), mouse anti-AP2 (1:100, ThermoFisher Scientific) and rabbit anti-endophilin 1 (1:200, Invitrogen), rabbit anti-dynamin 1 (1:150, Abcam), mouse anti-SNAP25 (1:500, Synaptic Systems) and mouse anti-syntaxin (1:500, Synaptic Systems). Imaging was similar to SpH imaging. mCherry fluorescence imaging was performed simultaneously to identify cells transfected with Cre/mCherry (Fig. 6a; Supplementary Fig. S14), or with wildtype CHC and mCherry (Supplementary Fig. S18a).

### Virus induction into hippocampal cultures

We modified the vector of adeno-associated virus (AAV)-GFP/Cre (Addgene# 49056) into AAV-mCherry/Cre, in which mCherry was attached at the C-terminal of Cre, and a nuclear localization sequence was tagged at the N-terminal of Cre. The constructed plasmid were sent to the Vigene Biosciences company for package into AAV-D/J serotype. This virus can be transduced into most (> 95%) cells.

### EM

Hippocampal cultures were fixed with 4% glutaraldehyde (freshly prepared, Electron microscopy sciences, Hatfield, PA) in 0.1 N Na-cacodylate buffer solution containing for at least one hour at 22–24 ^o^C, and stored in 4 °C refrigerator overnight. The next day, cultures were washed with 0.1 N cacodylate buffer, and treated with 1% OsO_4_ in cacodylate buffer for 1 hr on ice, and 0.25% uranyl acetate in acetate buffer at pH 5.0 overnight at 4 °C, dehydrated with ethanol, and embedded in epoxy resin. Thin sections were counterstained with uranyl acetate and lead citrate then examined in a JEOL200CX TEM. Images were collected with a CCD digital camera system (XR-100 from AMT, Danvers, MA) at a primary magnification of 10,000–20,000×. Synapses were selected based on the structural specialization including synaptic vesicle clustering, synaptic cleft and the postsynaptic density.

### Computational model

To determine the equilibrium shapes of $\Omega$-like membrane buds we used an elastic model. The system contains two distinct structural elements, the lipid bilayer and the clathrin coat covering the lipid bilayer at the bud base. The lipid bilayer was modeled as an elastic inextensible film exhibiting properties of a two-dimensional fluid, capable of sustaining a lateral tension, $\gamma$, and resisting the deformation of bending as described by Helfrich model^87^. The bilayer elastic energy, ${F}_{{\rm{m}}}$, was computed according to

1 $${F}_{{\rm{m}}}=\frac{1}{2}{\kappa }_{{\rm{m}}}\int {J}^{2}{dA}+\gamma A,$$

where $J$ is the local total curvature (twice mean curvature^88^) of the bilayer surface, $A$ is the bilayer area, ${\kappa }_{{\rm{m}}},$ is the bilayer bending modulus^87,89^. The first contribution to Eq.1 represents the bending energy upon vanishing spontaneous curvature^87^, whereas the second contribution is the thermodynamic work of the membrane area exchange with the surrounding plasma membrane subject to lateral tension, $\gamma$. The energy of the bilayer Gaussian curvature^87^ is not included in Eq.1 since the considered membrane deformations do not change the topological genus of the bilayer surface.

The clathrin coat is formed through cross-linking of protein complexes (triskelia)^90^. Therefore, in contrast to the lipid bilayer, the coat is not expected to have properties of a two-dimensional fluid and we considered it as a solid layer. In spite of a complex microstructure of the coat, we used a simplified approach and modelled it as a continuous layer of isotropic homogeneous elastic material characterized by Young modulus, $E$, and Poisson ratio, $\nu$ (ref. ^91^). The layer has an intrinsic stress-free shape of a sphere with radius ${R}_{S}$. The bending energy per unit area of such a layer can be written as^92^,

2 $${f}_{{\rm{C}}}={\kappa }_{C1}{\left({c}_{{\rm{m}}}+{c}_{{\rm{p}}}-2{R}_{S}^{-1}\right)}^{2}+{\kappa }_{C2}\left({c}_{{\rm{m}}}-{R}_{S}^{-1}\right)\left({c}_{{\rm{p}}}-{R}_{S}^{-1}\right),$$

where ${c}_{{\rm{m}}}$ and ${c}_{{\rm{p}}}$ are the two principle curvatures of the surface^88^, ${\kappa }_{C1}$ and ${\kappa }_{C2}$ are the bending elastic moduli of the coat, which can be expressed through $E$ and $\nu$, have opposite signs, ${\kappa }_{C1}\, >\, 0$ and ${\kappa }_{C2}\, <\, 0$ and similar absolute values^92^, so that we assumed ${\kappa }_{C1}=-{\kappa }_{C2}={\kappa }_{C}$. The elastic energy of the clathrin coat, ${F}_{C}$, is obtained by integration of ${f}_{{\rm{C}}}$ (Eq. 2) over the coat area.

To determine the equilibrium shape of an $\Omega$-bud for each extent of the clathrin coat polymerization on its base, we used the following procedure. We computationally created an initial coat-free $\Omega$-bud by application of a localized pulling force to the center of a flat circular region of lipid bilayer upon assumptions that the bilayer is subject to lateral tension, $\gamma$, and the boundary of the forming $\Omega$-bud base is set and fixed, as described in refs. ^9,13^. Then we imposed on the base of the initial $\Omega$-bud a clathrin coat with certain values of ${\kappa }_{C}$ and ${R}_{S}$ describing the desired phase of the coat polymerization. Based on our experimental observations (Fig. 2), the coat area was chosen to cover the whole base of the initial $\Omega$-bud from the boundary to the pore waist, and was assumed constant for all stages of the coat polymerization, hence, corresponding to the constant area scenario of clathrin coat formation^93^. The equilibrium $\Omega$-bud configuration was found by numerical minimization of the total energy, ${F}_{T}={F}_{{\rm{m}}}+{F}_{{\rm{C}}}$. The computations were performed using Ken Brakke’s Surface Evolver^94^.

### Data selection

For every cell recorded with a pipette under the whole-cell configuration, the data within the first 2 min at the whole-cell configuration were used, which avoided rundown of endocytosis (gradual disappearance of endocytosis) as previously reported under the whole-cell configuration for a long time^18,79^. For reconstructing R_fs+pre_, cells with less than 5 fusion events were not used, which avoided large fluctuations from individual cells.

STED images were analyzed with ImageJ and LAS X (Leica). Confocal images were analyzed with ImageJ and LAS X (Leica). The fluorescence intensity from an area covering the fluorescence spot was measured at every image frame. The full-width-half-maximum (W_H_) was measured from intensity profiles of 1–4 lines across the spot center.

### Statistical tests

Data were expressed as mean ± s.e.m. Replicates are indicated in results and figure legends. *n* represents the number of cells, fusion events or experiments as indicated in results and figure legends. The statistical test used is *t*-test or ANOVA. Although the statistics were performed based on the number of cells, fusion events and pre-close, each group of data were replicated from at least four primary chromaffin cell cultures. Each culture was from at least two glands from one bovine.

### Supplementary information

SUPPLEMENTAL MATERIAL
