# Supplementary material for: Clathrin mediates membrane fission and budding by constricting membrane pores
Source: Cell Discov. 2024 Jun 11;10:62. doi: 10.1038/s41421-024-00677-w (PMC11166961; doi:10.1038/s41421-024-00677-w)
Supplement: Supplementary file 1 — SUPPLEMENTAL MATERIAL [file 41421_2024_677_MOESM1_ESM.docx]

**Clathrin mediates membrane fission and budding by constricting membrane pores**

**Supplementary Information**


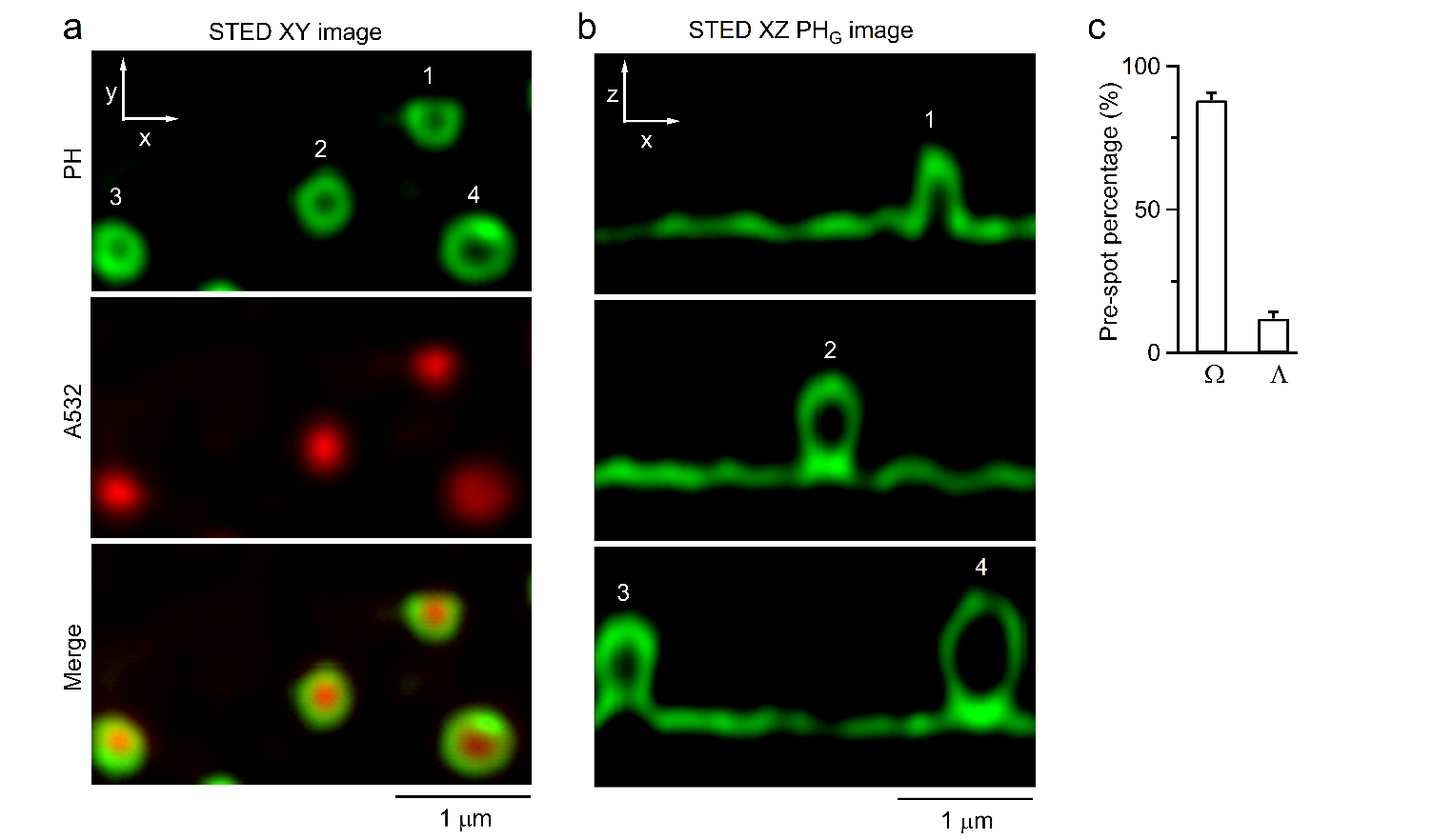


**Figure S1. Preformed spots (pre-spots) observed at the XY plane are mostly Ω-shape and sometimes Λ-shape**

**a,** PH_G_ and A532 images of four preformed PH_G_/A532 spots (pre-spots) at the STED XY-plane (merge image shown in the bottom). Labeled numbers correspond to those shown in panel b.

**b,** STED XZ image of the four pre-spots labeled in a.

**c,** The percentage of Ω and Λ for pre-spots observed at the XY-plane (n = 13 cells). Ω and Λ were identified with XZ-plane imaging, which was performed after XY-plane imaging.

This figure was taken from Ref. ^1^ with permission. For the convenience of readers, we replot this figure so that readers do not need to look for it in a different paper.

**
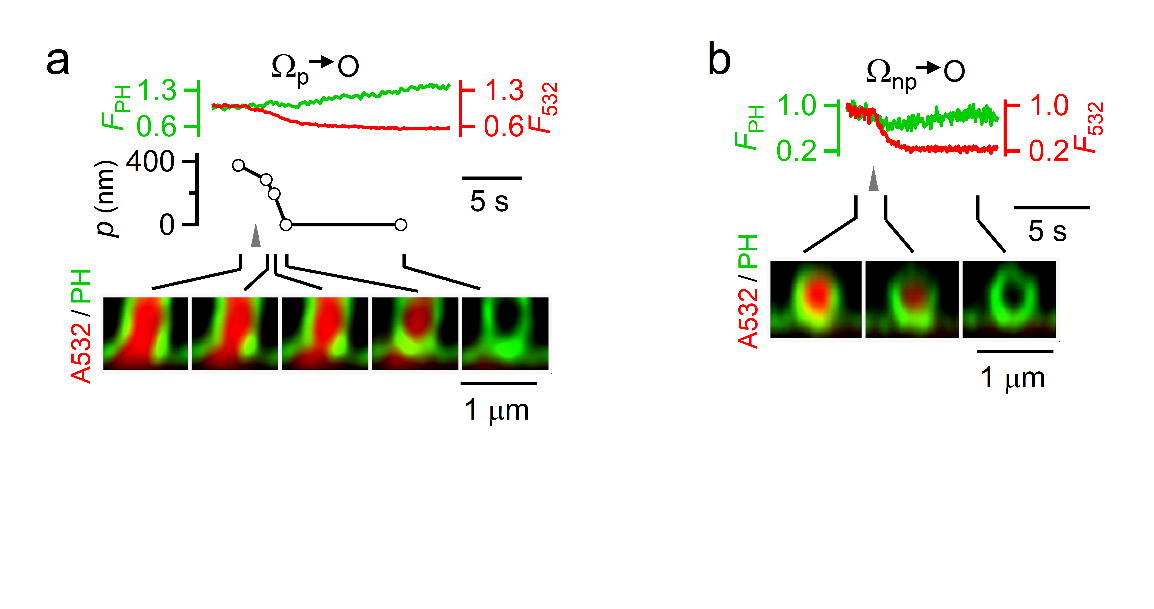
**

**Figure S2. STED XZ/Y_fix_ imaging reveals pre-close at the XZ-plane**

**a-b,** PH_G_ and A532 fluorescence (F_PH_, F_532_, normalized to baseline), and STED XZ/Y_fix_ images (at times indicated with lines) for a pre-Ω undergoing pore closure. a, a preformed-Ω (pre-Ω) with a visible pore (Ω_p_) that constricted and closed after depol_1s_ (gray triangle). b, a pre-Ω’ with a non-visible pore (Ω_np_) that closed after depol_1s_ (gray triangle); pore closure was detected as F_532_ bleaching while F_PH_ sustained or decayed with a delay. This figure was taken from Ref. ^1^ with permission. For the convenience of readers, we replot this figure so that readers do not need to look for it in a different paper.


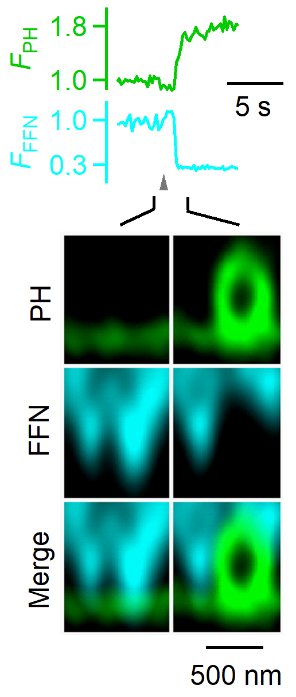


**Figure S3. Observation of FFN511 release and PH_G_-labelled fusion-generated Ω with STED XZ/Y_fix_ scanning**

F_PH_, FFN511 fluorescence (F_FFN_, normalized to baseline), and sampled images at times indicated with lines showing release of FFN511 and the appearance of PH_G_-labelled Ω-profile due to diffusion of PH_G_ from the plasma membrane into the fusion-generated Ω-profile (fs-Ω).


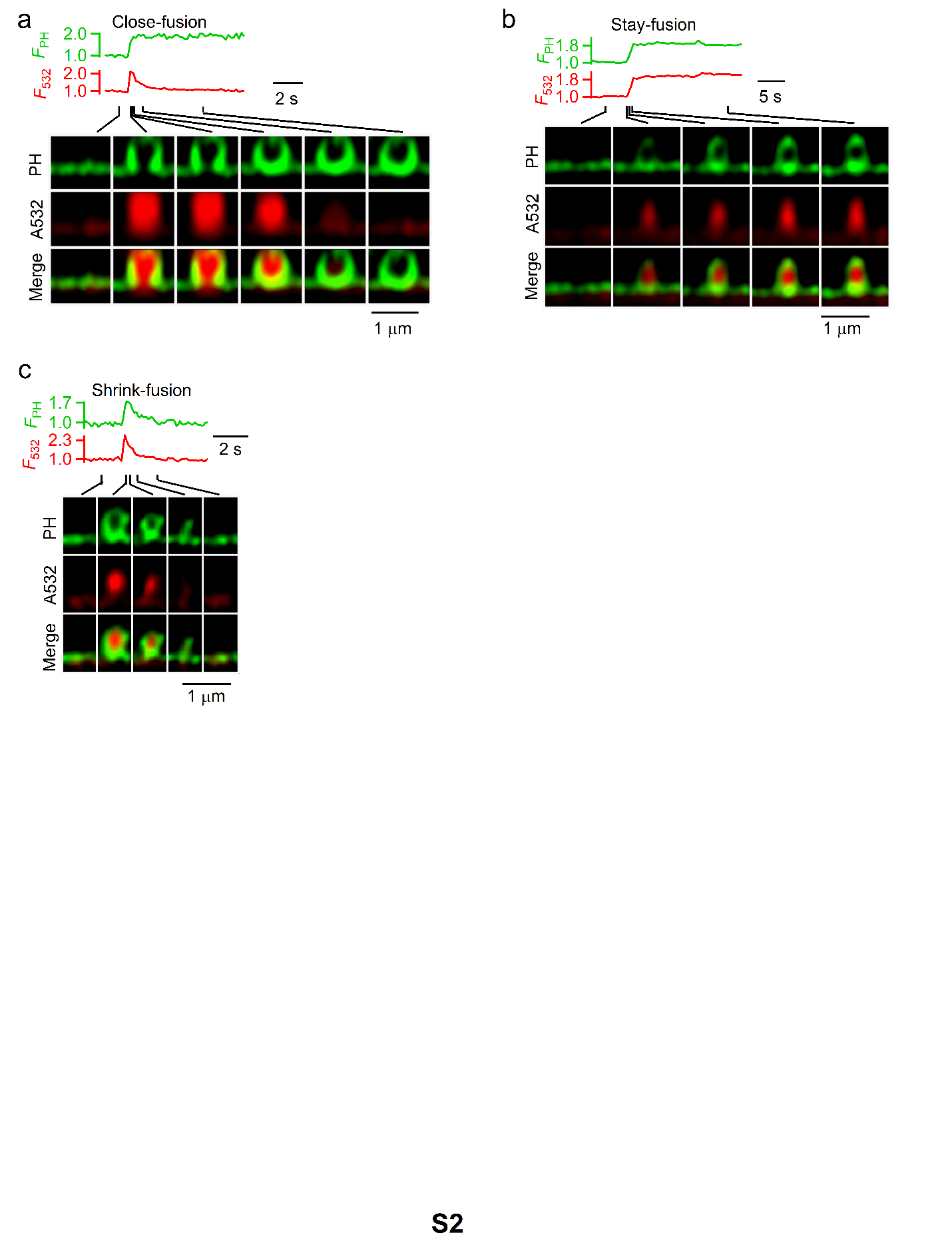


**Figure S4. Fusion-generated Ω-profiles may close its pore, maintain an open pore or shrink to merge with the plasma membrane**

**a-c,** PH_G_ fluorescence (F_PH_, normalized to baseline), A532 spot fluorescence (F_532_, normalized to baseline), and sampled images at times indicated with lines showing that fusion-generated Ω-profiles close its pore (a, close-fusion), maintain an open pore (b, stay-fusion) or shrink to merge with the plasma membrane (c, shrink-fusion). Fusion was detected as the sudden appearance of PH_G_-labelled Ω-profiles with a A532 spot within a single XZ/Y_fix_ imaging frame (every 26-200 ms)^2^. Panel a is taken from Ref. ^2^ with permission; panels b and c are taken from Ref. ^3^ with permission. For the convenience of readers, we replot these published figures so that readers do not need to look for them in different papers.


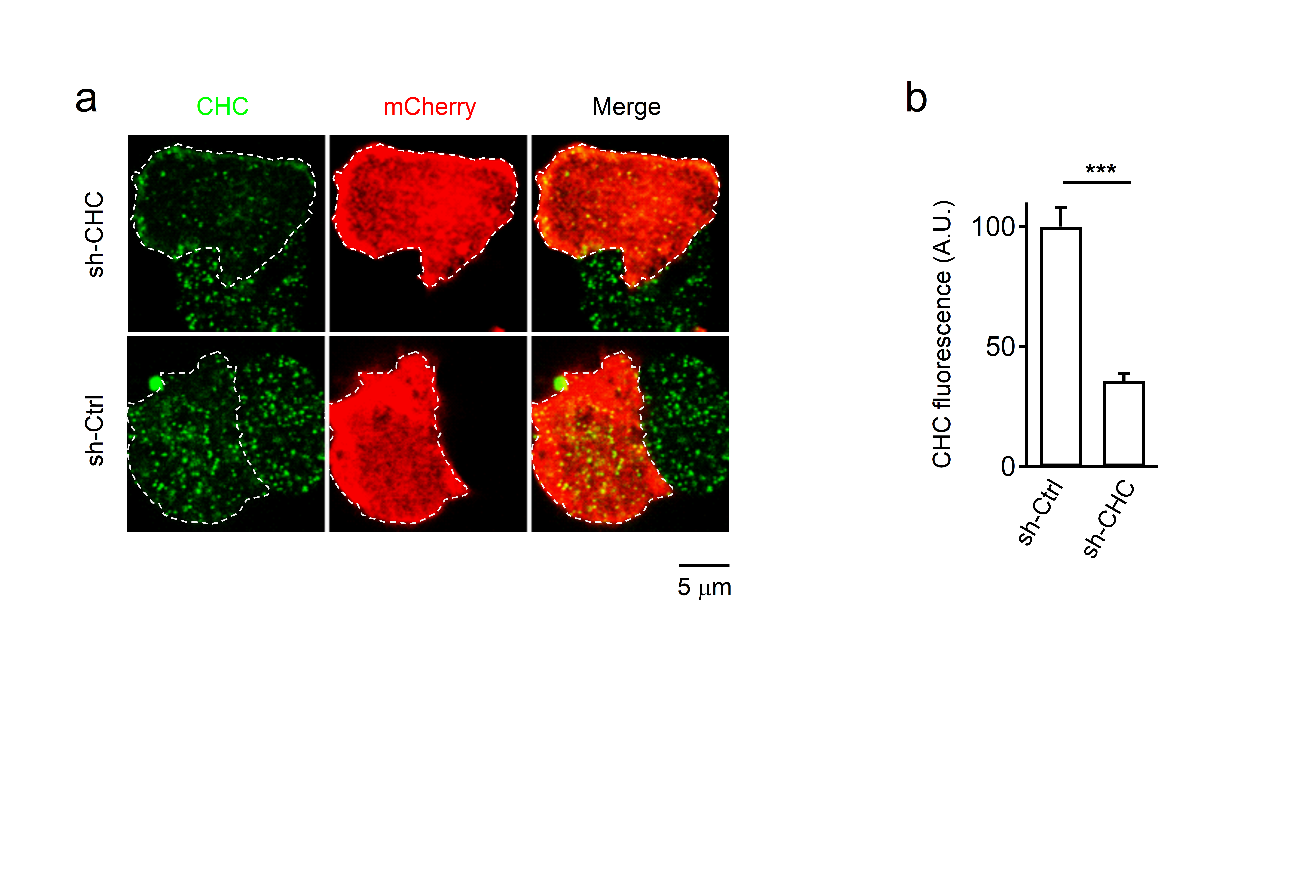


**Figure S5. Sh-CHC transfection reduces clathrin expression in chromaffin cells**

**a,** **Left:** clathrin antibody labelling of two cells, one transfected with a plasmid containing mCherry tagged sh-CHC or sh-Ctrl (outlined with dotted lines; also see mCherry image in the middle panels), the other without transfection of shRNA plasmid (see no-mCherry image in the middle panels).

**Middle:** mCherry images showing transfection with mCherry tagged sh-CHC or sh-Ctrl plasmids (also outlined with dotted lines).

**Right:** Merged images. Cells were immunostained and imaged in 3 days after mCherry tagged sh-CHC or sh-Ctrl transfection.

**b,** The fluorescence intensity of clathrin puncta labeled with clathrin antibody for cells transfected with sh-CHC (10 cells) or sh-Ctrl (10 cells The fluorescence intensity was normalized by adjacent untransfected cells). Cells were imaged in 3 days after the procedure of transfection was carried out.


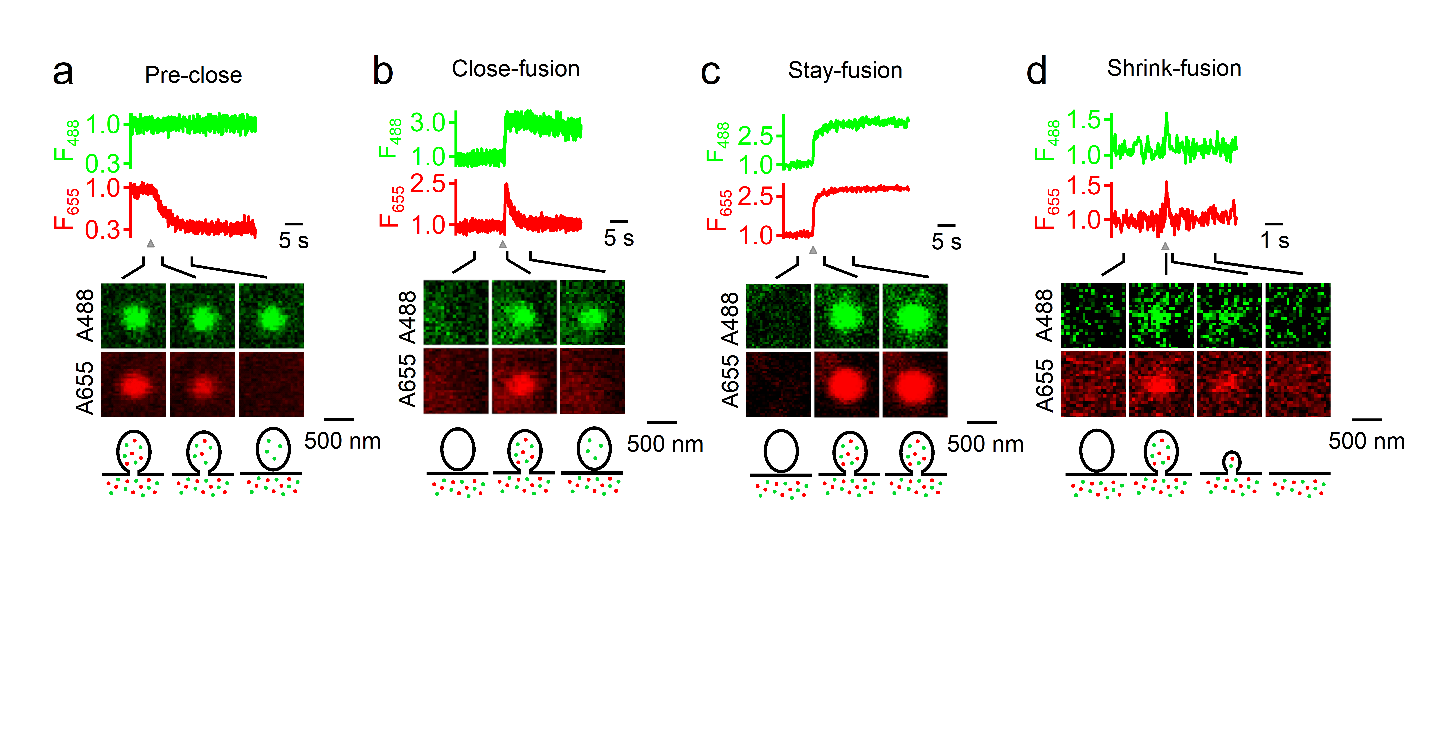


**Figure S6. Detection of pre-spot closure, close-fusion, stay-fusion, and shrink-fusion by confocal XY-plane imaging with Atto 655 and Alexa 488 in the bath**

**a,** Atto 655 (A655) fluorescence (F_655_, strongly excited), Alexa 488 (A488) fluorescence (F_488_, weakly excited), and confocal XY/Z_fix_ images showing pre-spot pore closure (pre-spot-close). Pre-spot-close was detected as F_655_ decay while F_488_ sustained, due to pore closure that prevents bleached A655 (by strong excitation) from exchanging with the fluorescent A655 in the bath. A schematic drawing of pre-spot-close is also presented in the bottom. Detection of pore closure with imaging of A655 (excited strongly) and A488 (excited weakly) was characterized systematically in Refs. ^2-4^.

**b-d,** Atto 655 (A655) fluorescence (F_655_, strongly excited), Alexa 488 (A488) fluorescence (F_488_, weakly excited), and confocal XY/Z_fix_ images showing close-fusion (b), stay-fusion (c), and shrink-fusion (d). A schematic drawing of the corresponding fusion mode is also presented in the bottom. Close-fusion was detected as F_655_ decay while F_488_ sustained, due to pore closure that prevents bleached A655 (by strong excitation) from exchanging with the fluorescent A655 in the bath. The sudden appearance of A655 and A488 spot (fluorescence reaching the peak within ~200 ms) was used to detect fusion. Detection of fusion modes and fusion pore closure with imaging of A655 (excited strongly) and A488 (excited weakly) was verified with concurrent confocal or STED imaging of NPY-EGFP release or FFN511 release, and with STED imaging of the sudden appearance of (within single frame: ~26-200 ms) of PH_G_-labelled Ω containing A532 spot or releasing FFN511^2-4^.

**
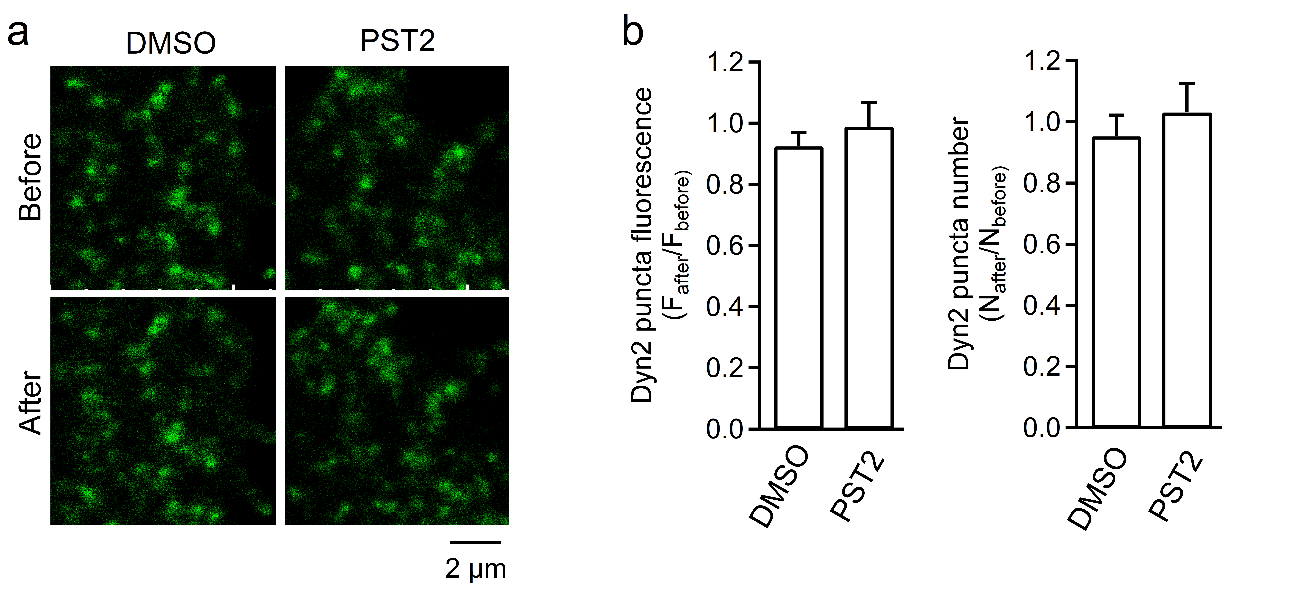
**

**Figure S7. Pitstop 2 does not affect dynamin2-mNeonGreen puncta fluorescence intensity or number**

**a,** Dynamin 2-mNeonGreen images at the cell-bottom membrane of chromaffin cells transfected with dynamin2-mNeonGreen. Images were captured before and 10 minutes after bath application of DMSO (0.3%, left) or pitstop 2 (PST2, 30 μM, including 0.3% DMSO).

**b,** Left: the ratio of dynamin 2 (Dyn2) puncta fluorescence intensity after and before application (F_after_/F_before_) of DMSO (13 cells, 2 cultures) or pitstop 2 (PST2, 15 cells, 2 cultures). No significant difference was observed. Each culture from 3 adrenal glands.

Right: similar to the left, except that puncta fluorescence intensity (F_after_/F_before_) is replaced with puncta number (N_after_/N_before_): no significant difference was observed. Cells are the same as in the left.


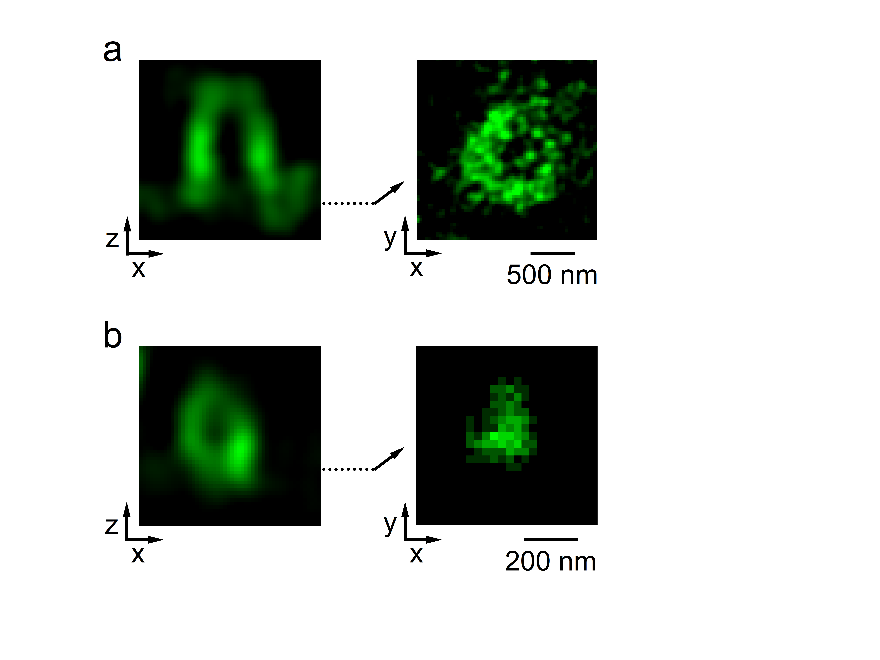
**Figure S8. Confocal imaging of PH-mScarlet-labeled pre-**Ω **with a visible or non-visible pore**

Confocal images of PH-mScarlet-labelled pre-Ω with a visible pore (a) or a non-visible pore (b) at the XZ-plane (left) or the XY-plane with a Z-focal plane across the pore region (right). The pre-Ω in panels a and b are the same as those shown in Fig. 2h_I_ and Fig. 2h_II_, respectively.

**Figure S9. Identifying the apparently tilted membrane invagination**

**a,** Platinum replica EM images showing an oval-shape membrane structures oriented in parallel to the top-to-down view direction – a complete oval/round-shape with bright and clear edge. Left: the EM image.

Right: the same EM image as on the left, but with a drawing (green) along the clear and bright oval/round-shape outline.

**b,** Similar arrangement as in panel a, but showing an apparently tilted membrane invagination – an inversed U-shape bright and clear edge (see the green drawing on the right) plus the remaining non-bright and non-clear edge (see the red drawing on the right) that appeared to be connected with the plasma membrane.


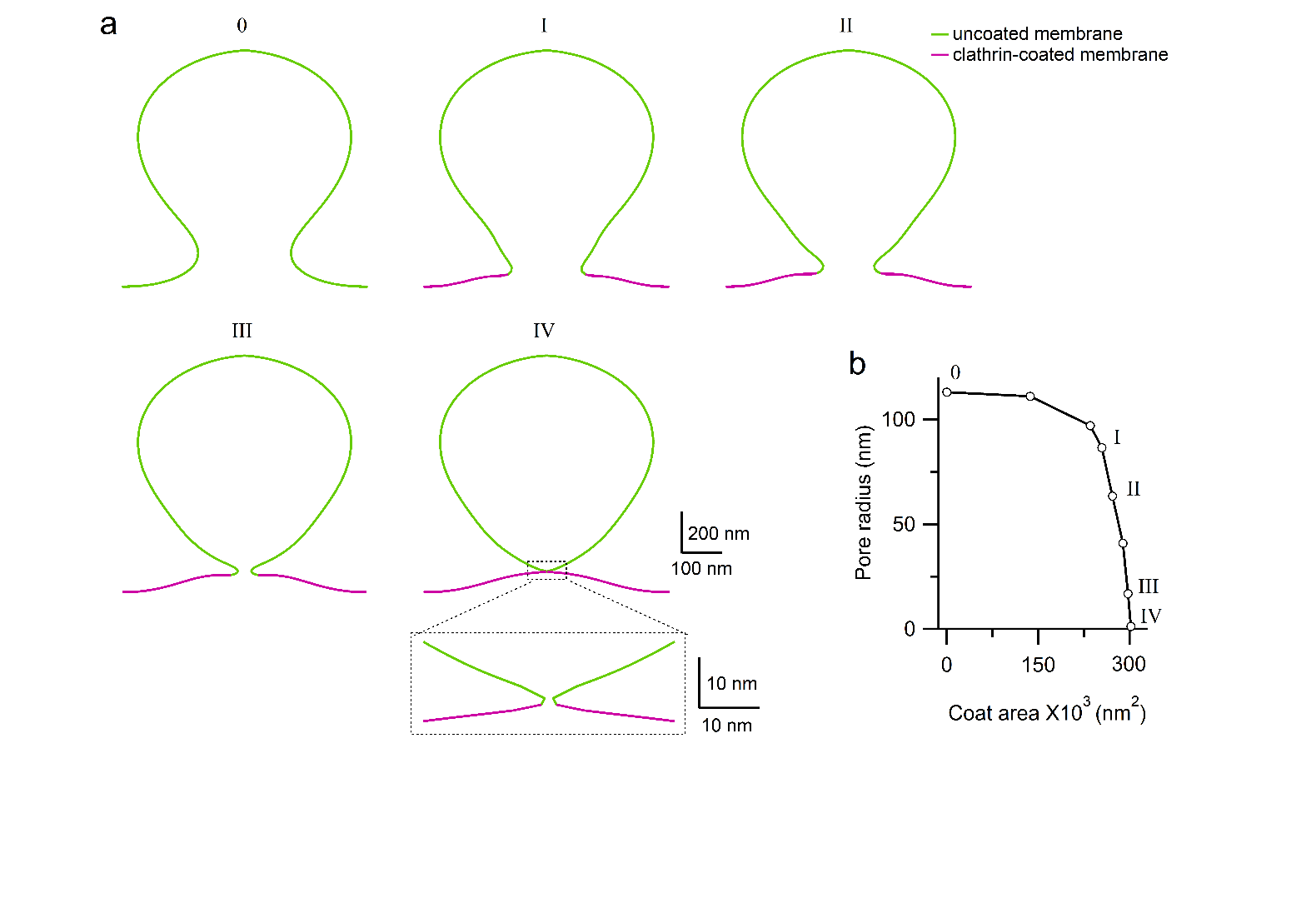


**Figure S10. Evolution of** $\boldsymbol{\Omega}$**-bud shape (pore waist) as the area of the clathrin coat increases**

**a,** Evolution of Ω-bud shape (Ω-bud 0, I, II, II and IV) as clathrin coat area increases from 0 (Ω-bud 0) to 30,000 nm^2^ (Ω-bud IV) at the Ω-bud’s base (coated membrane: magenta; uncoated membrane: green). The coat area in Ω-bud 0, I, II, III and IV can be found in panel b’s data points with corresponding labels. Ω-bud IV inset: boxed area enlarged. Under the constant curvature scenario for the coat polymerization at the Ω-bud base, the coat is assumed to have constant values of the bending modulus $\kappa_{C}=2\kappa_{B}=40\text{k}_{\text{B}}\text{T}$ (Ref. ^5^) and intrinsic radius of the curvature, $R_{S}=70\text{nm}^{2}$, which corresponds to a typical dimension of clathrin coated vesicles. The area of the coat increases describing a gradual propagation of the coat from the boundary towards the waist of the Ω-bud pore.

**b,** Pore waist radius plotted versus clathrin coat area at the bud base. Data points labelled with 0, I, II, III, or IV correspond to Ω-buds with the same label in panel a.


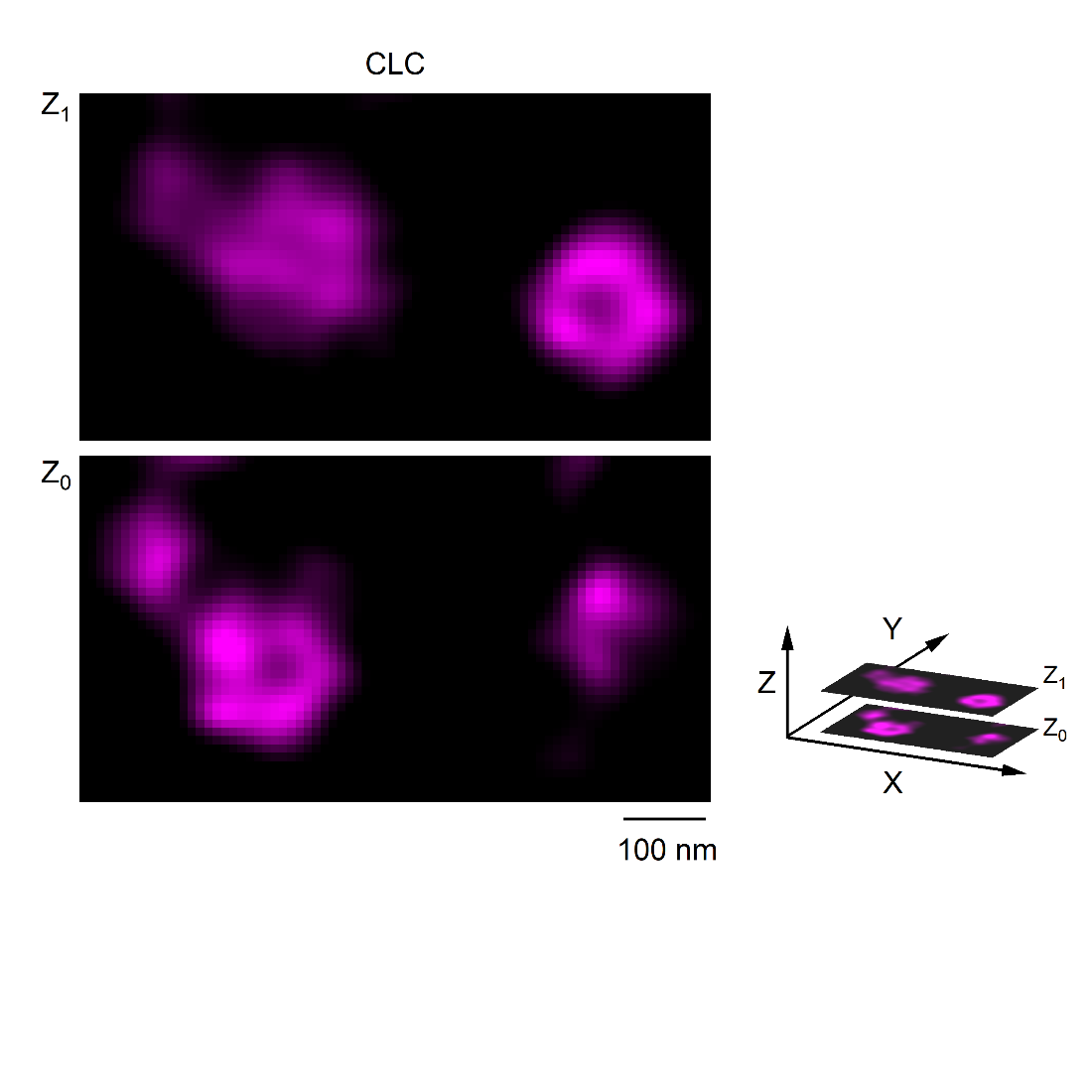


**Figure S11. Clathrin ring at the XY-plane may not be detected at a fixed Z-location**

STED XY-plane image of SNAP-CLC-siR-647 at two Z-locations (Z0 and Z1) 180 nm apart showing rings observed at one Z-location (e.g., ring at the right of Z1) may not appear as rings in the other Z-location (e.g., structure at the right of Z0). Images were obtained with a higher-spatial-resolution STED scope equipped with a high-power 775-nm depletion laser.


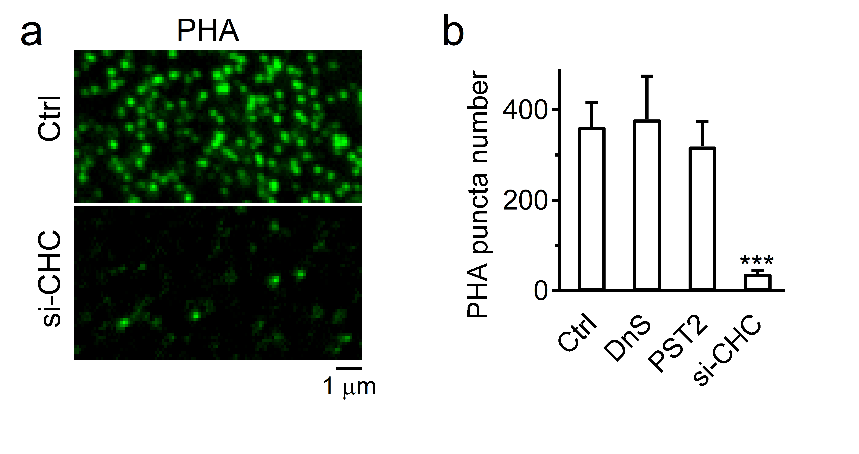


**Figure S12. Transfection of clathrin heavy chain siRNA (si-CHC) reduces the percentage of PHA_G_ puncta in chromaffin cells**

**a,** Confocal XY-plane images of PHA_G_ at the cell bottom membrane in control (Ctrl, upper) and in a cell transfected with si-CHC (lower).

**b,** The average number of PHA_G_ puncta in cells in control (Ctrl, 24 cells), in the presence of dynasore (DnS, 14 cells, 80 μM, bath), pitstop (PST2, 16 cells, 30 μM, bath), and si-CHC (12 cells). ***: p < 0.001 (t test).

**
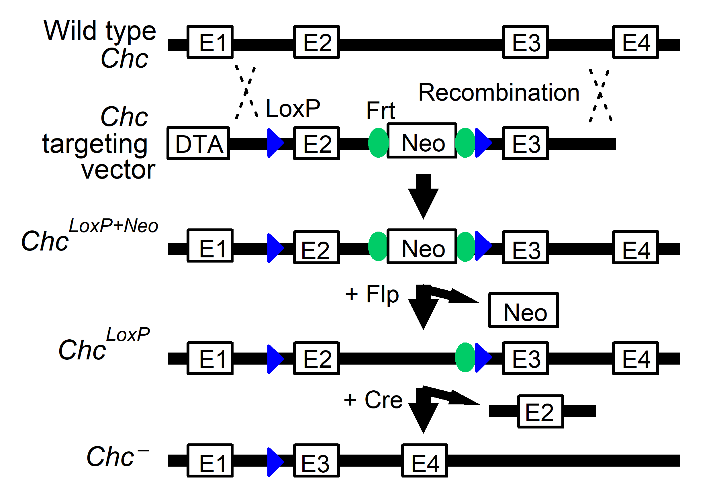
**

**Figure S13. Procedure for generating CHC conditional knockout mouse**

Wild-type *Chc*: endogenous Chc contains E1, E2, E3, and E4 exons. *Chc* targeting construct: A 7.7 kb *Chc* genomic DNA fragment containing E2 and E3 was used as the homologous arm, and was subcloned at the *SacII* and *NotI* sites of the cloning vector containing a negative selection marker, diphtheria toxin A gene (DTA). A loxP site was inserted at 538 bp upstream of E2 and a Frt-loxP-flanked Neomycin (Neo) cassette was inserted at 640 bp downstream of E2 for positive selection. *Chc^LoxP+Neo^*: The *Chc* targeting construct was linearized and electroporated into G4 embryonic stem (ES) cells as described previously^6^. Targeted ES cells were selected by Neo selectant, G418, and identified by Southern blot. Targeted ES cells were injected into C57BL/6J blastocysts implanted in ICR females, giving rise to mouse chimeras. Chimeric mice were bred with C57BL/6J mice to generate *Chc* targeted germline mice (*Chc^Neo+LoxP^*). *Chc^LoxP^*: Crossing *Chc^Neo+LoxP^* mice with ROSA26-FLPe mice (Jackson Laboratory) removed the FRT-flanked Neo gene in off springs, leading to generation of *Chc* conditional knockout (*Chc^Loxp^*) mice. *Chc*^−^: introducing the Cre recombinase deletes E2 and creates a premature termination codon in E3, giving rise to the *Chc* null (*Chc*^−^) allele.

**
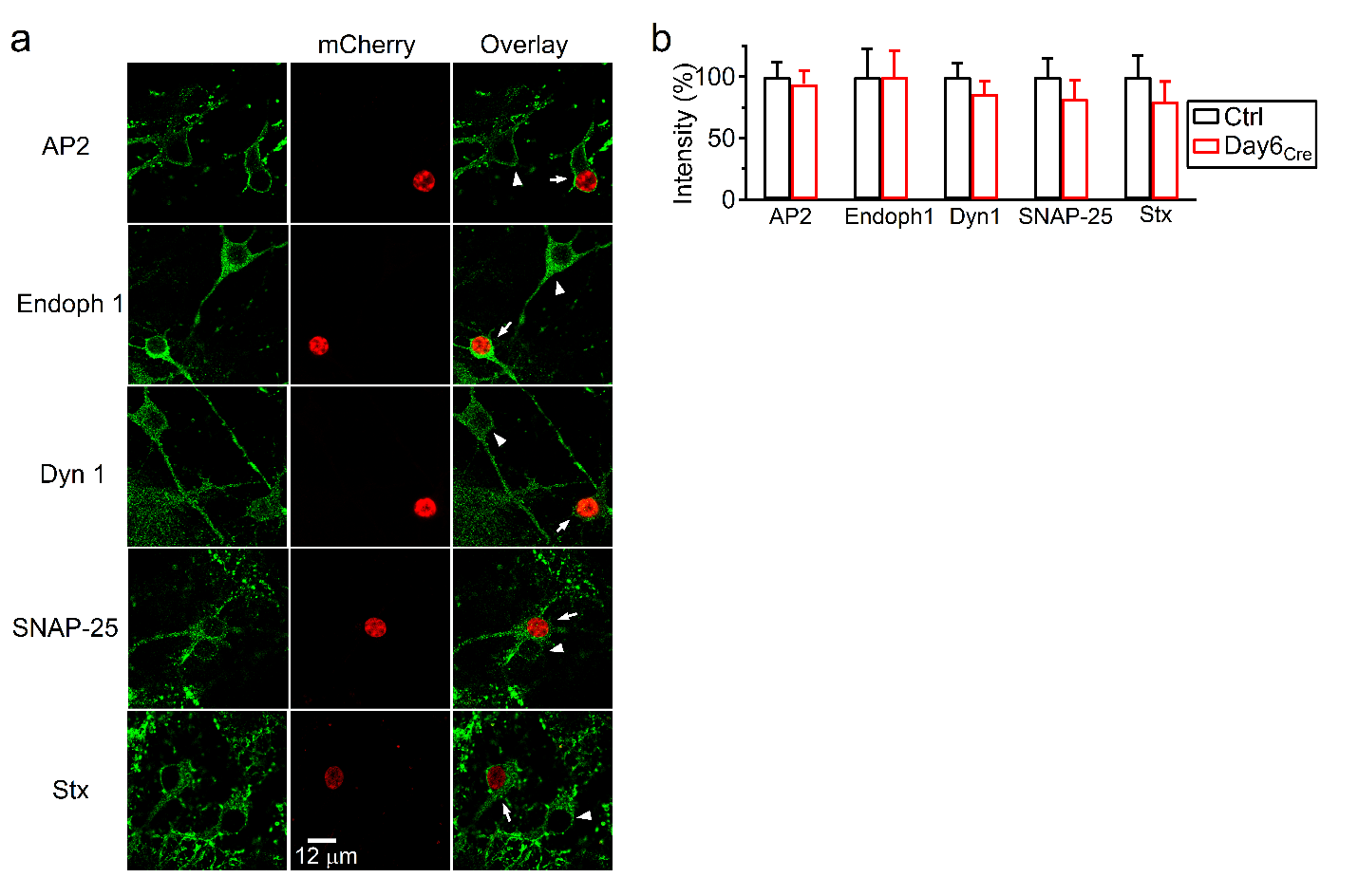
**

**Figure S14. Expression of proteins associated with endocytosis and exocytosis does not change at Day6_Cre_ hippocampal cultures**

**a,** Immunostaining of AP2, endophilin 1 (endoph 1), dynamin 1 (Dyn 1), SNAP-25, syntaxin (Stx) (green) at 6 days after Cre/mCherry transfection to *Chc^loxp/loxp^* hippocampal neurons (red: mCherry fluorescence indicates transfected cells, arrows). Green and red images are superimposed in the right. Neighboring neurons without Cre/mCherry transfection are also shown as control (triangles).

**b,** Immunostaining intensity of AP2, endophilin 1 (Endoph 1), dynamin 1 (Dyn 1), SNAP-25, syntaxin (Stx) in neurons transfected with Cre/mCherry for 6 days (Day6_Cre_) and in neighboring neurons without Cre/mCherry transfection (Control). Immunostaining intensity was measured from the soma. Data were normalized to the corresponding control mean value. Each bar was taken from 15-25 neurons from 3 transfections.

**
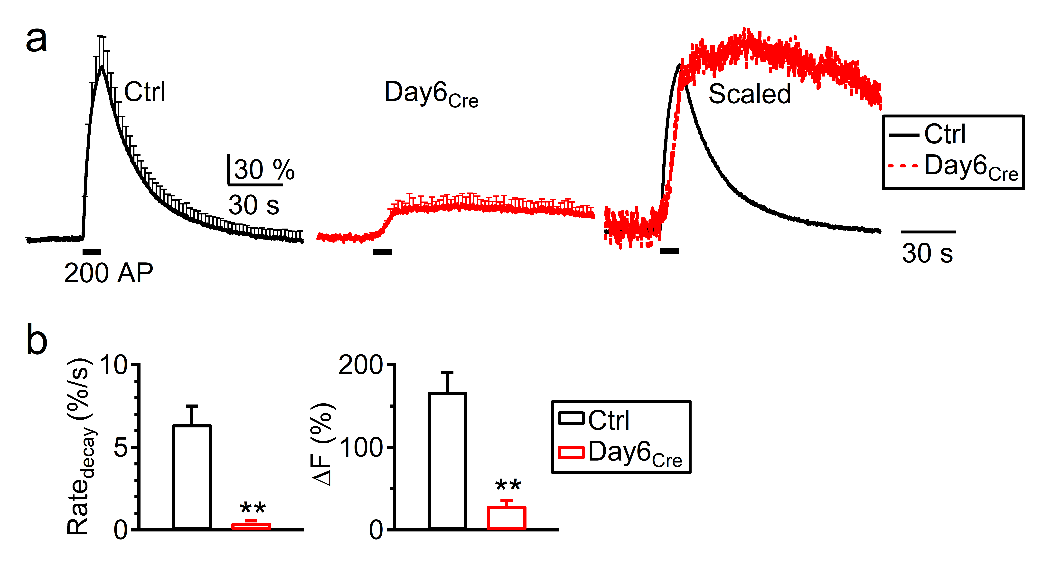
**

**Figure S15. Inhibition of endocytosis is observed when Cre is transfected with a calcium-phosphate transfection procedure**

**a-b,** SpH fluorescence **(**F_SpH_) trace (a, mean + s.e.m.), Rate_decay_ (b, mean + s.e.m.) and ΔF (b, mean + s.e.m.) induced by AP_20H/10s_ in Ctrl (n = 7) or in Day6_Cre_ cultures (n = 4). In both cultures, Cre was transfected with the calcium-phosphate transfection procedure^7^. a: Mean F_SpH_ in Day6_Cre_ is also scaled (dash red) for comparison with F_SpH_ in control. b: Rate_decay_ was measured from normalized F_SpH_ trace where the normalized ΔF was 100% (%/s, percentage of the normalized ΔF decay per second). Ctrl: *Chc^Loxp/Loxp^* synapses transfected with SpH alone for 6 days. *, p < 0.05; **, p < 0.01 (t test).


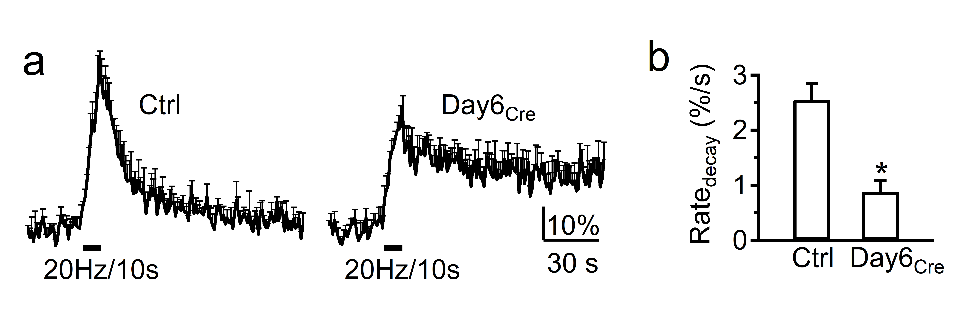


**Figure S16. Imaging of synaptobrevin-pHluorin also reveals inhibition of endocytosis at Day6_Cre_ cultures**

Synaptobrevin-pHluorin fluorescence traces (F_SbpH_, mean + s.e.m.) induced by AP_20H/10s_ in Ctrl (n = 4) and Day6_Cre_ (n = 4) hippocampal synapses at 34-37^o^C are shown.

**
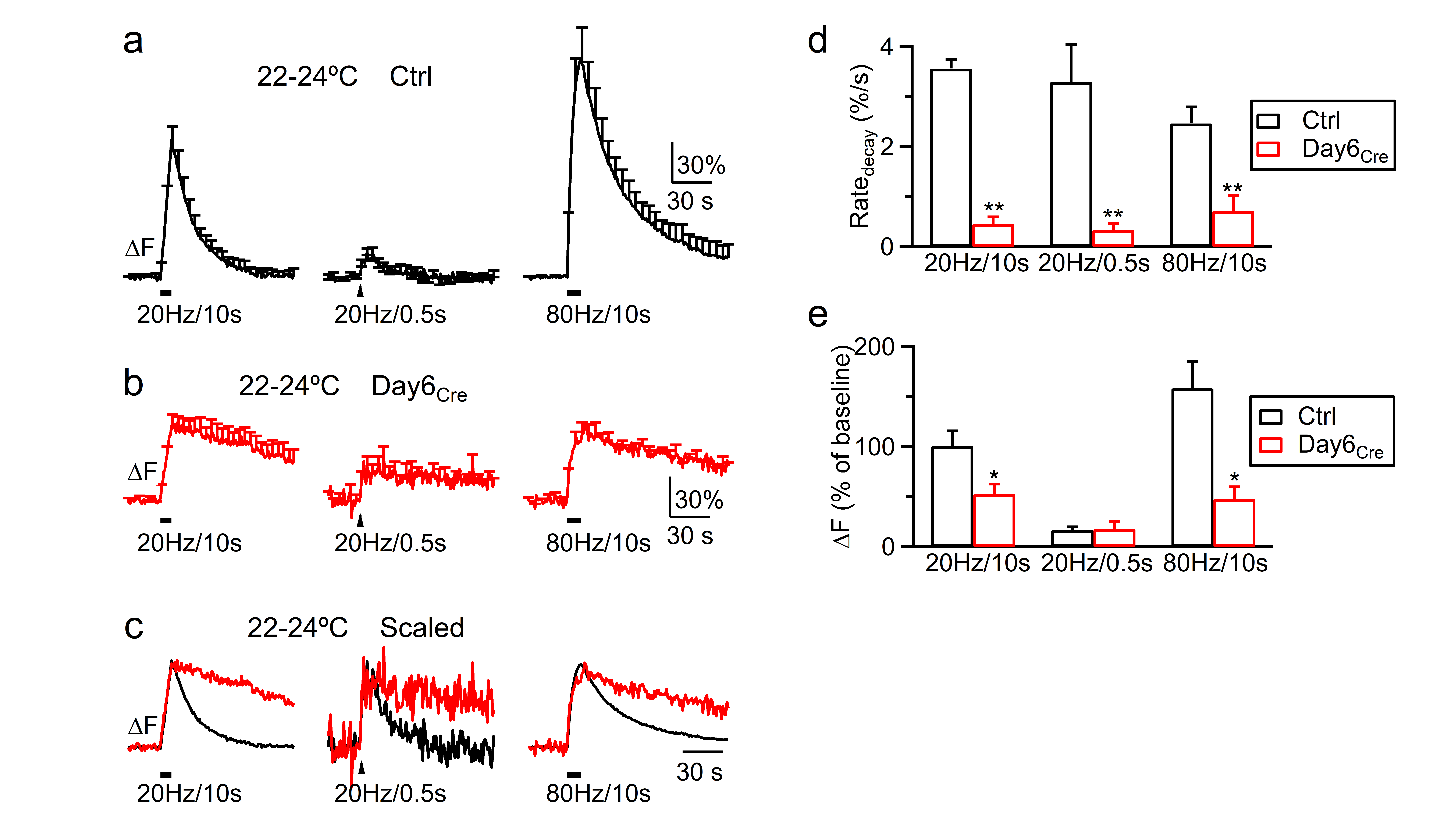
**

**Figure S17. Inhibition of F_SpH_ decay after AP trains at Day6_Cre_ synapses at 22-24^o^C**

**a-b,** F_SpH_ (mean + s.e.m, every 5 s) induced by AP_20H/10s_, AP_20H/0.5s_ and AP_80H/10s_ at 22-24^o^C in control (a, Ctrl) and Day6_Cre_ culture (b).

**c,** Mean F_SpH_ in panel a-b (same color code) superimposed and scaled.

**d-e,** Rate_decay_ (d) and ΔF (e) induced by AP_20H/10s_, AP_20H/0.5s_ and AP_80H/10s_ at 22-24^o^C in Ctrl and Day6_Cre_ culture (mean + s.e.m). d: Rate_decay_ was measured from normalized F_SpH_ trace where the normalized ΔF was 100% (%/s, percentage of the normalized ΔF decay per second); experimental numbers labeled above each bar. e: ΔF is the percentage (%) of F_SpH_ increase over the baseline. Ctrl: *Chc^Loxp/Loxp^* synapses transfected with SpH alone for 6 days. *, p < 0.05; **, p < 0.01.

**
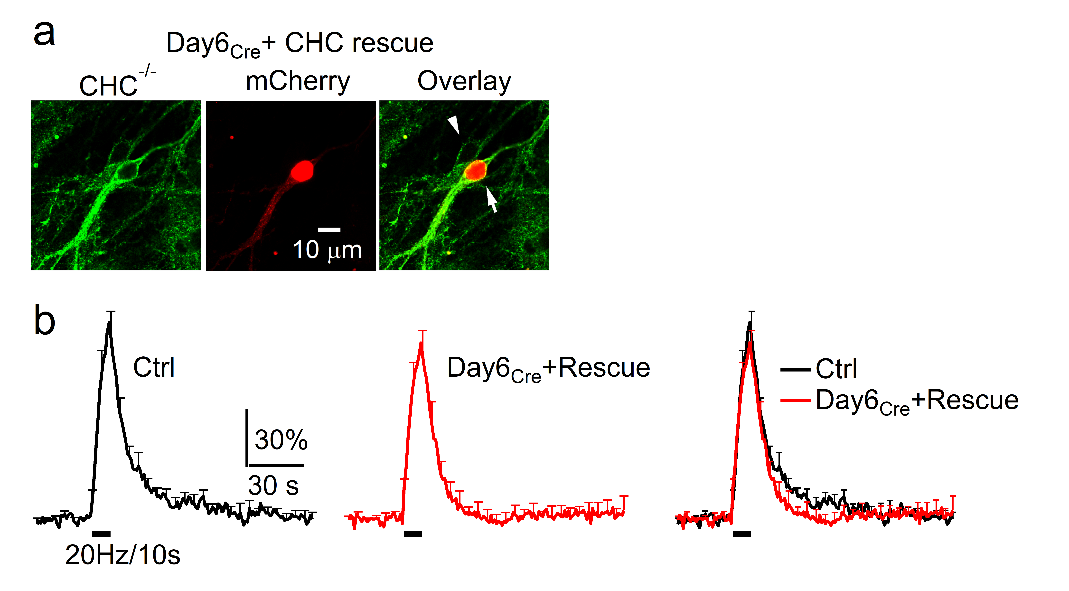
**

**Figure S18. Inhibition of F_SpH_ decay at Day6_Cre_ is rescued by wild-type clathrin overexpression**

**a,** CHC antibody staining and mCherry fluorescence at 6 days after transfection of Cre, mCherry and wild-type Chc to *Chc^Loxp/Loxp^* culture (Day6_Cre_+Rescue). Arrow: mCherry-positive cell (Chc overexpression rescue Chc reduction). Triangle: mCherry-negative cell.

**b,** F_SpH_ trace induced by AP_20Hz/10s_ in Ctrl (n = 9) and Day6_Cre_+Rescue culture (n = 5) at 34-37^o^C (Right: left and middle superimposed).

**
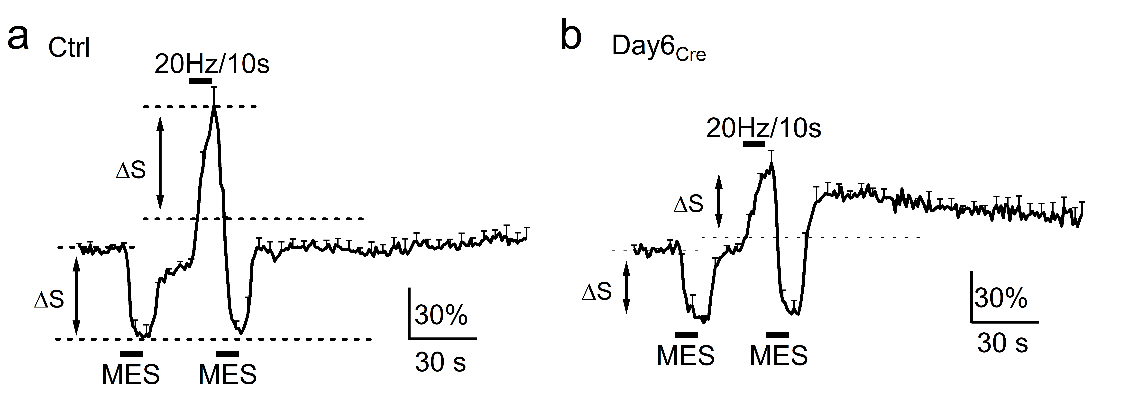
**

**Figure S19. Inhibition of F_SpH_ decay at Day6_Cre_ is not caused by the block of re-acidification, but by inhibition of endocytosis**

**a-b,** The MES solution (pH:5.5, bars) quenched F_SpH_ to a similar level (lower dotted line) before and 2 s after AP_20Hz/10s_ in Ctrl (n = 8, a, p = 0.74, paired t test) and Day6_Cre_ (n = 9, b, p =0.81, paired t test) synapses at 34-37^o^C, suggesting that F_SpH_ decay reflect primarily endocytosis of SpH molecules at the plasma membrane. ΔS represents the fluorescence of SpH molecules at the plasma membrane that can be quenched by MES solution before stimulation. The upper dotted line is the expected value, if slower F_SpH_ decay is due to slower re-acidification following endocytosis.

**References**

1 Shin, W. *et al.* Preformed Omega-profile closure and kiss-and-run mediate endocytosis and diverse endocytic modes in neuroendocrine chromaffin cells. *Neuron* **109**, 3119-3134 e3115 (2021).

2 Shin, W. *et al.* Visualization of Membrane Pore in Live Cells Reveals a Dynamic-Pore Theory Governing Fusion and Endocytosis. *Cell* **173**, 934-945 (2018).

3 Shin, W. *et al.* Vesicle Shrinking and Enlargement Play Opposing Roles in the Release of Exocytotic Contents. *Cell Rep* **30**, 421-431 e427 (2020).

4 Chiang, H. C. *et al.* Post-fusion structural changes and their roles in exocytosis and endocytosis of dense-core vesicles. *Nat. Commun* **5**, 3356 (2014).

5 den Otter, W. K. & Briels, W. J. The generation of curved clathrin coats from flat plaques. *Traffic* **12**, 1407-1416 (2011).

6 George, S. H. *et al.* Developmental and adult phenotyping directly from mutant embryonic stem cells. *Proc. Natl. Acad. Sci. U. S. A* **104**, 4455-4460 (2007).

7 Kononenko, N. L. *et al.* Clathrin/AP-2 Mediate Synaptic Vesicle Reformation from Endosome-like Vacuoles but Are Not Essential for Membrane Retrieval at Central Synapses. *Neuron* **82**, 981-988 (2014).
